# KMT2A degradation is observed in decitabine‐responsive acute lymphoblastic leukemia cells

**DOI:** 10.1002/1878-0261.13792

**Published:** 2025-01-04

**Authors:** Luisa Brock, Lina Benzien, Sandra Lange, Maja Huehns, Alexandra Runge, Catrin Roolf, Anett Sekora, Gudrun Knuebel, Hugo Murua Escobar, Christian Junghanss, Anna Richter

**Affiliations:** ^1^ Department of Medicine, Clinic III – Hematology, Oncology, Palliative Medicine Rostock University Medical Center Germany; ^2^ Institute of Pathology Rostock University Medical Center Germany

**Keywords:** acute leukemia, decitabine, DNMT1, KMT2A, menin, revumenib

## Abstract

Hypermethylation of tumor suppressor genes is a hallmark of leukemia. The hypomethylating agent decitabine covalently binds, and degrades DNA (cytosine‐5)‐methyltransferase 1 (DNMT1). Structural similarities within DNA‐binding domains of DNMT1, and the leukemic driver histone‐lysine *N*‐methyltransferase 2A (KMT2A) suggest that decitabine might also affect the latter. In acute lymphoblastic leukemia (ALL) cell lines, and xenograft models, we observed increased *DNMT1*, and *KMT2A* expression in response to decitabine‐induced demethylation. Strikingly, KMT2A protein expression was diminished in all cell lines that experienced DNMT1 degradation. Moreover, only cells with reduced KMT2A protein levels showed biological effects following decitabine treatment. KMT2A wild‐type, and rearranged cells were locked in G2 and G1 cell cycle phases, respectively, likely due to p27/p16 activation. Primary sample gene expression profiling confirmed different patterns between KMT2A wild‐type, and translocated cells. This newly discovered decitabine mode of action via KMT2A degradation evokes anti‐leukemic activity in adult ALL cells, and can act synergistically with menin inhibition. Following the successful clinical implementation of decitabine for acute myeloid leukemia, the drug should be considered a potential promising addition to the therapeutic portfolio for ALL as well.

## Graphical abstract

We demonstrate that decitabine (DEC) not only degrades the DNA methyltransferase DNMT1 but also the leukemic driver lysine methyltransferase KMT2A likely due to structural similarity of the DNA‐binding CXXC domains. DEC influences KMT2A downstream processes and synergizes with menin inhibitor revumenib (REV) to decrease leukemic cell proliferation, and cell cycle progression. Created in BioRender. https://BioRender.com/q61r396.

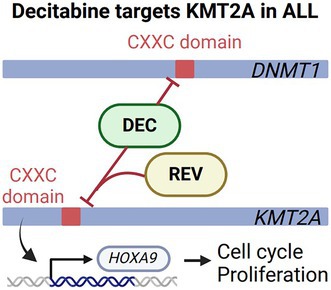

**Abbreviations**
ALL: acute lymphoblastic leukemia
AML: acute myeloid leukemia
CXXC: Cys‐Glu‐x‐Cys‐x‐x‐Cys motif
DEC: decitabine
DNMT1: DNA (cytosine‐5)‐methyltransferase 1
KMT2A: histone‐lysine *N*‐methyltransferase 2A
KMT2A‐r: KMT2A‐rearranged
KMT2A‐wt: KMT2A wild type
PBMC: peripheral blood mononuclear cells
PDX: patient‐derived xenograft
REV: revumenib
siRNA: small interfering RNA
TSG: tumor suppressor gene

## 1 Introduction

Adult acute leukemias are severe conditions with 5 year overall survival rates between 20% and 40% [1, 2]. Several mechanisms of pathogenesis have been described; one of them being intense hypermethylation of tumor suppressor genes (TSG) [3, 4]. CpG island methylation is mediated via DNA methyltransferases like DNMT1 which therefore attribute to TSG inactivation, disease initiation, and progression in acute leukemia [5]. DNMT1 recognizes, and binds unmethylated DNA via its CXXC domain [6]. This motif is highly conserved, consists of a Cys‐Glu‐x‐Cys‐x‐x‐Cys core structure, and is required for protein function, mediating the enzymatic activity of the methyltransferase [7].

The cytosine analogon decitabine (DEC) is incorporated during DNA replication. In contrast to cytosine, it cannot be methylated, thus resulting in demethylation, transcription, and reactivation of silenced TSG [8, 9]. In addition, DEC covalently binds DNMT1 via the CXXC domain, and induces its proteasomal degradation [10]. In the clinical setting, DEC is successfully used for the treatment of acute myeloid leukemia (AML), and myelodysplastic syndromes [11, 12]. Promising results were also achieved in acute lymphoblastic leukemia (ALL) cases [13, 14, 15]. In preclinical studies, DEC was also shown to reduce tumor cell proliferation, and induce apoptosis in acute leukemia [16, 17, 18], but the regulatory mechanisms behind those effects are not completely understood.

Besides DNMT1, other proteins also contain DNA‐recognizing CXXC domains. The lysine methyltransferase KMT2A possesses a highly conserved N‐terminal CXXC motif which binds CpG containing structures [19]. KMT2A activates cell proliferation via HOXA9, and MEIS1 upregulation and is therefore a major player in leukemogenesis [20, 21, 22]: roughly every second acute leukemia is characterized by aberrantly increased *HOXA9* expression [23, 24]. Several fusion proteins are described in acute leukemia, mainly with other epigenetic or transcriptional regulators like AFF1, MLLT1, or MLLT3, accounting for strongly divergent epigenomic, and gene expression patterns in KMT2A‐rearranged (KMT2A‐r) leukemia compared to other cytogenetic entities [25, 26]. During replication, KMT2A fusion proteins recruit the super elongation complex, and positive transcription elongation factor b toward the *HOXA9* locus. The histone methyltransferase DOT1L, which is together with KMT2A fusion partner AFF1 part of the super elongation complex, then methylates lysine residue 79 of histone 3, and facilitates extensively increased *HOXA9* expression as a main mechanism of KMT2A‐r‐induced pathogenicity [23].

The cell cycle progression can also be influenced by KMT2A, which regulates cyclin‐dependent kinase inhibitors p16^*INK4A*^, p18^*INK4C*^, and p27^*KIP1*^ [27, 28]. Interestingly, erasing the KMT2A CXXC domain, and inserting the DNMT1 CXXC sequence instead did not interfere with *HOXA9* regulation, and other KMT2A protein tasks, including leukemogenesis *in vivo* [29].

In view of the structural similarities between DNMT1, and KMT2A we investigated if DEC is able to not only degrade DNMT1 but also KMT2A, possibly revealing a previously unknown DEC‐induced anti‐tumor mechanism. Indeed, our results demonstrate KMT2A protein degradation as well as inhibition of cell cycle progression following DEC incubation in ALL cell lines, and xenograft models. Further, our experiments elucidate synergistic anti‐leukemic potential of double KMT2A blockade using DEC and the menin inhibitor revumenib. This finding suggests that given the high expression, and importance of KMT2A in ALL, DEC alone or in combination should be considered for clinical use in this entity as well.

## 2 Materials and methods

### 2.1 Cell lines and exposure experiments

Human B—ALL cell lines SEM (RRID:CVCL_0095), RS4;11 (RRID:CVCL_0093), REH (RRID:CVCL_1650), and NALM‐6 (RRID:CVCL_0092) as well as human AML lines MV4;11 (RRID:CVCL_0064), and MOLM13 (RRID:CVCL_2119) were purchased from DSMZ (Braunschweig, Germany), and maintained at 37 °C and 5% CO_2_ in IMDM medium (SEM), Alpha MEM medium (RS4;11), or RPMI medium (REH, NALM‐6, MV4;11, MOLM13) supplemented with 10% heat‐inactivated fetal calf serum, and 100 μg·mL^−1^ penicillin/streptomycin (all PAN—biotech, Aidenbach, Germany). Medium was replaced twice weekly, and cells were seeded at a density of 3.3 × 10^5^ cells·mL^−1^ for further culturing or inhibitor experiments. Cells were regularly checked for authenticity (cell surface flow cytometry), and mycoplasma contamination. Decitabine was purchased from Selleck Chemicals (Munich, Germany), and dissolved in DMSO (cell culture experiments) or saline (*in vivo* studies). Revumenib was obtained from MedCemExpress (Monmouth Junction, NJ, USA), and reconstituted in DMSO.

### 2.2 Methylation analysis

DNA isolation was carried out using the NucleoSpin™ tissue kit (Macherey‐Nagel, Düren, Germany) following the manufacturer's guidelines. The EpiTect Bisulfite Kit (Qiagen, Hilden, Germany) was used for bisulfite conversion. Global methylation was assessed by methylation‐specific LINE‐1 qPCR as previously described [30]. *HOXA9* and *MEIS1* CpG promoter methylation was analyzed by pyrosequencing using a Q24 Pyromark instrument (Qiagen), and commercially available assays Hs_MEIS1_01_PM PyroMark CpG assay and Hs_HOXA9_03_PM PyroMark CpG assay. In both assays, 2.5 μL of the diluted 10× PCR primer sets, and 50 ng of bisulfite treated DNA were used in a 25 μL reaction volume and annealing temperature of 58 °C. The sequencing reaction was carried out using 5 μL of the PCR product, and 2 μL of the 10× sequencing primer according to the manufacturer's recommendations. Target CpGs were evaluated by pyromark q24 2.0.7 software (Qiagen).

### 2.3 Western blot

Protein expression and H3K4 trimethylation was assessed by western blot as previously described [31]. Antibodies, solvents, and dilutions are listed in Table S1.

### 2.4 Gene expression analysis

RNA isolation and cDNA synthesis were carried out using the RNeasy® Mini kit (Qiagen), and PrimeScript™ RT Reagent Kit (Takara Bio Europe, Saint‐Germain‐en‐Laye, France), respectively. Gene expression analysis was performed in technical duplicates or triplicates with 25 ng cDNA, SensiFast™ Probe Lo‐ROX master mix (Bioline, London, UK), and manually designed TaqMan primers and probes (Table S2) in a ViiA7 Real‐Time PCR system (Thermo Fisher Scientific, Waltham, MA, USA). Reactions were performed at 50 °C for 2 min, 95 °C for 10 min, and followed by 40 cycles of 15 s at 95 °C and 1 min at 60 °C. All mean *C*_T_ values were normalized to the respective *GAPDH* mean *C*_T_, and changes in gene expression following inhibitor incubation were calculated using the $2^{-\Delta \Delta C_{T}}$ formula.

### 2.5 Intracellular flow cytometry

Cells were harvested and washed in cold PBS, fixed in methanol‐free 4% formaldehyde for 15 min, and washed twice in PBS before permeabilization in ice‐cold 90% methanol for 30 min. After two steps of PBS washing, cells were blocked in antibody dilution buffer for 10 min, and incubated with 2 μg KMT2A primary antibody (Bio‐Techne, Wiesbaden Nordenstadt, Germany), for 35 min at room temperature. Cells were again washed twice in antibody dilution buffer, and analyzed using the FACSLyric™ device (BD, Heidelberg, Germany) with facsuite™ software (BD; version 1.0.6.5230).

### 2.6 Cell viability, vitality and cytotoxicity assays

Cell proliferation was assessed by trypan blue dye exclusion, and metabolic activity was analyzed using WST‐1 assay (Roche, Mannheim, Germany) as previously described [31]. Apoptosis and cell cycle analysis using Annexin V (BD)/propidium iodide staining were carried out as previously described [31]. To assess healthy cell toxicity, erythrocyte lysis assay as well as peripheral blood mononuclear cell isolation, and viability were performed as previously described [32].

### 2.7 Ki67 immunofluorescence staining

Cytospin preparations [31] were fixed in ice‐cold methanol for 10 min and permeabilized/blocked in 0.5% triton X‐100, and 2% BSA for 1 h before incubation with 1 : 50 diluted Ki67 antibody (PE/Cyanine7 anti‐mouse/human Ki‐67 Antibody, Biolegend, San Diego, CA, USA) over night. Slides were counter‐stained using Roti®Mount FluorCare DAPI solution (Roth, Karlsruhe, Germany), and imaged with a Axio Vert.A1 with Colibri 5 light source (Zeiss, Jena, Germany), and Axiocam 305 mono camera (Zeiss).

### 2.8 siRNA‐mediated gene knockdown

For gene knockdown, cell lines SEM and NALM‐6 were transfected with pools of two different DsiRNAs targeting either *DNMT1* or *KMT2A* (150 nm of each DsiRNA; hs.Ri.DNMT1.13.1/hs.Ri.DNMT1.13.2 or hs.Ri.KMT2A.13.1/hs.Ri.KMT2A.13.2, respectively); or non‐targeting negative control DsiRNA (all Integrated DNA Technologies, Coralville, IA, USA). Cells were electroporated in serum‐free medium at 300 V and 950 μF using a Gene Pulser Xcell system with CE Module, and ShockPod™ (Bio‐Rad, Feldkirchen, Germany). The silencing effect was evaluated by gene expression analysis using probe‐based qPCR 24 h after electroporation.

For inhibitor studies following siRNA application, cells were first electroporated, then placed in the incubator for 24 h, and afterward incubated with 100 nm DEC or DMSO (control) for another 72 h.

### 2.9 Morphology assessment

Cell morphology in response to inhibitor treatment was analyzed by Pappenheim staining of 10^5^ cells per cytospin as previously described [31].

### 2.10 *In vivo* model systems, treatment procedures and study endpoints

All animal experiments were approved by the review board of the federal state Mecklenburg‐Vorpommern, Germany (reference number: LALLF MV/7221.3‐1.1‐002/15), and animals were bred, housed and observed as previously described [16]. Primary human patient samples were collected at the Rostock University Medical Center (2008–2020) in accordance to the Declaration of Helsinki, and approved by the local ethics committee of the Rostock University Medical Center (A 2018‐0122). All participants gave written informed consent. Clinical, and molecular patient characteristics are available in Table S3. *In vivo* tumor cell injection into mouse tail veins, monitoring of blast counts using peripheral blood (PB) flow cytometry (GFP^+^ or CD45^+^/CD19^+^), and bioluminescence imaging were performed as previously described [16, 30, 33, 34].

For cell line‐derived xenograft model systems (SEM–fluc, RS4;11–fluc), nine NOD scid gamma (NSG; Charles River Laboratories, Sulzfeld, Germany) animals each were treated with either DEC or saline for 4 days starting 7 days after tumor cell injection. DEC was dissolved in isotonic saline, and applied intraperitoneal at a concentration of 0.4 mg·kg^−1^ body weight. Only mice with a bioluminescence signal indicating successful tumor cell engraftment were included in experiments. Randomization was performed based on sex, age, weight, and bioluminescence imaging signal intensity at day 7 after cell injection. Study groups were not blinded to the investigators. Mice were euthanized by narcotization (75 mg·kg^−1^ ketamine, 5 mg·kg^−1^ xylazine) followed by cervical dislocation 30 days after tumor cell injection. For patient‐derived xenograft (PDX) models, 3–4 mice per PDX model were injected with cells derived from each patient. One to two mice each were treated with either DEC or saline as described above. Data resulting from these experiments describing the effect of DEC on blast proliferation have previously been published [16].

### 2.11 Analysis of previously published gene and protein expression data

Affymetrix Human Genome U133 Plus 2.0 Array data of a total of 71 KMT2A‐r, and 102 KMT2A wild‐type ALL patients were retrieved from the GEO database (accession numbers GSE79533, GSE66006, GSE26281, GSE28497, GSE12995; [35, 36, 37, 38, 39]), and analyzed with the GEO2R tool.

Proteomic data of ALL cell lines from the FORALL Functional Omics Resource of Acute Lymphoblastic Leukemia was used to assess protein expression in KMT2A‐r, and wild‐type samples [40, 41].

### 2.12 Statistical analyses

All values are expressed as mean ± standard deviation (SD). Gaussian normality distribution was tested in all cases using Kolmogorov–Smirnov test, determining the following parametric or non‐parametric with *post hoc* test. The exact test is indicated in the respective figure legends. No statistical analysis was conducted when groups contained less than three individual values. Statistical analyses were performed using graphpad prism software (version 8; GraphPad Software, Boston, MA, USA). Statistical significance was defined as *P < 0.05, **P < 0.005, and ***P < 0.001. Synergy was calculated according to the Bliss independence model [42] with positive values indicating synergy whereas negative Bliss scores suggest antagonism.

## 3 Results

### 3.1 DEC induces similar effects in DNMT1 and KMT2A

**Fig. 1:**
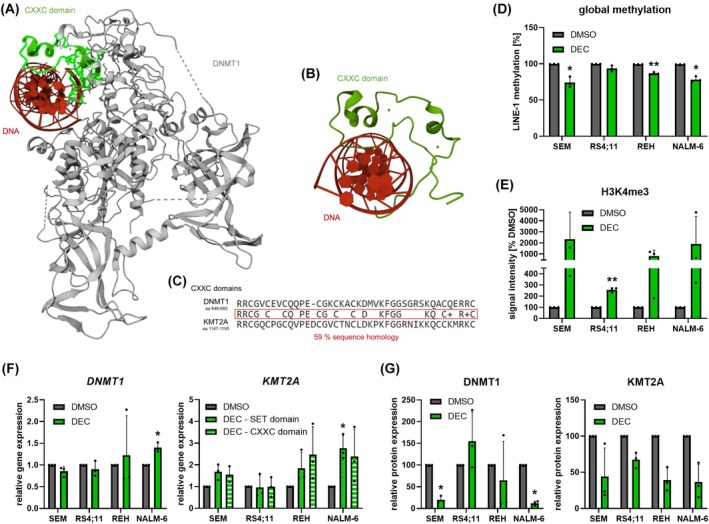
Decitabine (DEC)‐induced effects on methyltransferase activity, gene and protein expression. (A) 3D crystal protein structure of DNMT1 (gray) in complex with DNA (red). The DNA‐binding CXXC domain is colored in green. RCSB Protein data bank PDB identifier 3PTA [6, 74]. (B) Crystal structure of the KMT2A CXXC domain (green) bound to human DNA (red). RCSB Protein data bank PDB identifier 2KKF [75, 76]. (C) Amino acid (aa) sequences of the DNMT1 (top), and KMT2A (bottom) CXXC domains were retrieved from uniprot [77]. Shared amino acids were analyzed using blastp suite‐2sequences [78] search, and depicted in the middle box. (D–G) Acute B‐lymphoblastic leukemia cell lines SEM, RS4;11, REH, and NALM‐6 were incubated with 1 μm DEC or vehicle substance (DMSO) for 72 h. (D) Influence on global methylation was analyzed by methylation‐specific qPCR of the LINE‐1 retrotransposon. *n* = 3, mean ± SD; paired *t* test. (E) Quantitative analysis of histone 3, lysine residue 4 trimethylation (H3K4me3) by western blot was used to determine the global transcription rate. *n* = 3, mean ± SD; ratio paired *t* test of absolute GAPDH‐normalized signal intensities. (F) Probe‐based gene expression analysis of *DNMT1*, and *KMT2A*. Both, the N‐terminal CXXC‐domain as well as the C‐terminal SET domain have been analyzed. *n* = 3, mean ± SD; paired *t* test of Δ*C*_t_ vs. *GAPDH* values. (G) Quantitative analysis of DNMT1, and KMT2A protein expression. *n* = 3, mean ± SD; ratio paired *t* test of absolute GAPDH‐normalized signal intensities. *P < 0.05; **P < 0.01.

Methyltransferases DNMT1 and KMT2A both harbor a CXXC domain. Using publically available 3D crystal structures of the CXXC domains of DNMT1 (Fig. 1A), and KMT2A (Fig. 1B) bound to DNA, we observed a strong structural similarity between these methyltransferases. Mapping the amino acid sequences of the CXXC domains demonstrated 59% sequence homology (Fig. 1C).

During DNA replication, DEC is incorporated into the DNA strand instead of cytosine but cannot be methylated. We therefore checked the global DNA methylation in four B‐cell precursor ALL cell lines following DEC incubation. Reduced methylation values were observed in all cell lines (Fig. 1D), resulting in increased genome‐wide transcription rates (Fig. 1E). The degree of demethylation positively correlated with the rise in histon H3 lysin 4 trimethylation, a marker of overall transcription: Cell lines SEM, and NALM‐6 demonstrated the highest changes in both analyses while RS4;11 cells were least affected.

Decitabine also covalently binds and thus degrades DNMT1. We therefore evaluated its expression after DEC incubation, elucidating stable or mildly elevated *DNMT1* gene expression (Fig. 1F) while the expression of the DNMT1 protein was significantly decreased in SEM, and NALM‐6 cells (Fig. 1G). Again, and comparable to the results of *DNMT1*, *KMT2A* gene expression was slightly increased. Of note, this effect was present independently of the concentration used in the experiments, ranging from low (100 nm) to above‐clinical levels (1000 nm) (Fig. S1A). This transcriptional upregulation was observed in both *KMT2A* SET, and CXXC domains. Intriguingly, probably due to the structural homology between CXXC domains, DEC also degraded the KMT2A protein in a range of concentrations (Fig. 1G, Fig. S1B). Of note, this decline in KMT2A expression was only observed following DEC incubation and not with other substances known to induce cytotoxic cell death, pointing out that the observed degradation effect is not a general mechanism of cellular stress (Fig. S1C).

In summary, the structural similarity of the DNA‐binding domains of DNMT1, and KMT2A suggests a comparable degradation of both methyltransferases following DEC incubation.

### 3.2 DEC influences KMT2A downstream targets HOXA9 and MEIS1

**Fig. 2:**
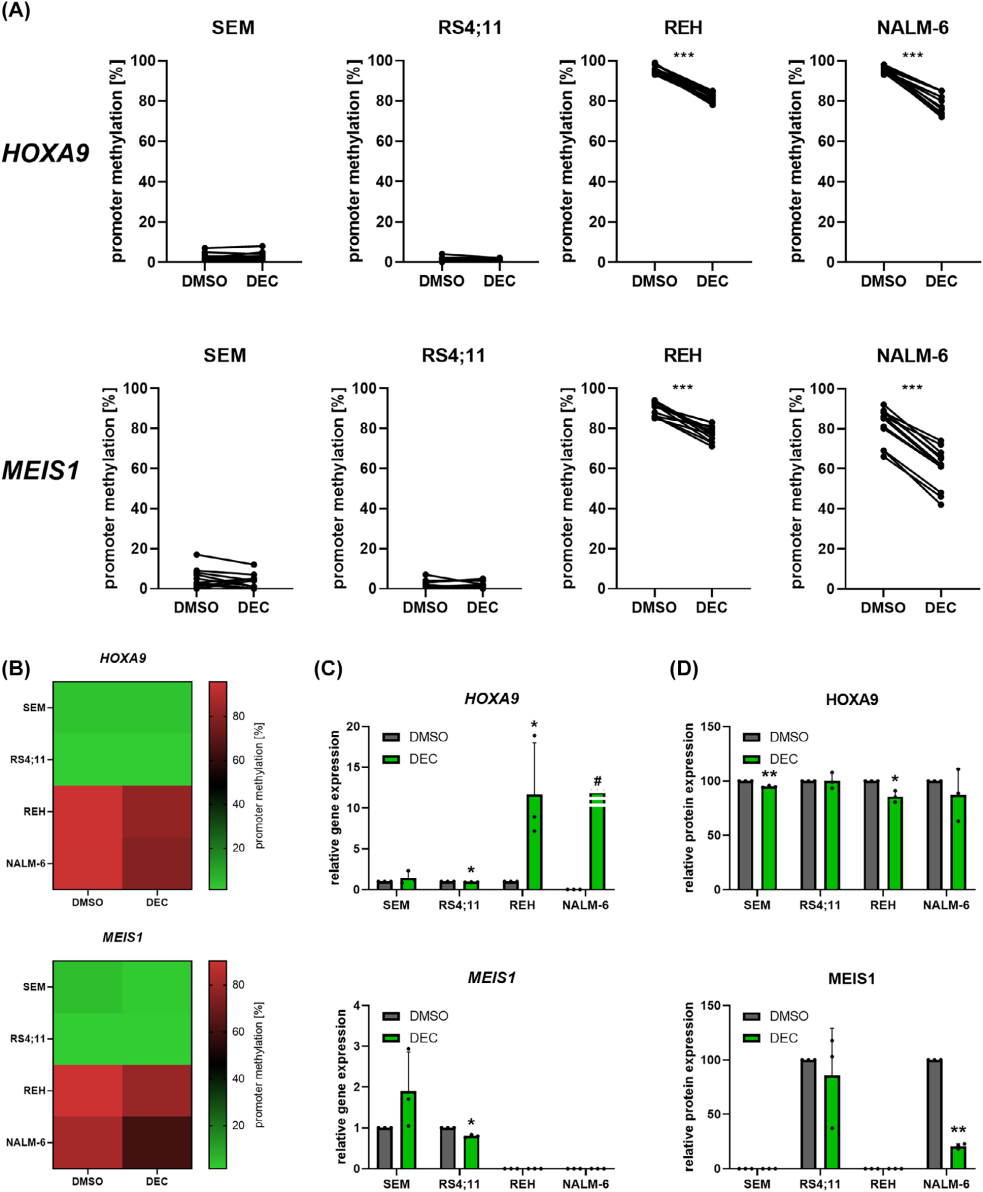
Decitabine (DEC; 72 h, 1 μm)‐induced effects on HOXA9 and MEIS1 in acute B‐lymphoblastic leukemia cell lines SEM, RS4;11, REH and NALM‐6. (A) Analysis of the promoter methylation using PyroMark assays. Six (*HOXA9*) or four (*MEIS1*) CpG islands were evaluated, and the change in absolute methylation of three biological replicates is displayed. Wilcoxon matched‐pairs signed rank test. (B) Heatmap displaying the overall promoter methylation of cells incubated with vehicle (DMSO) or DEC. (C) Probe‐based gene expression analysis. ^#^No *HOXA9* gene expression could be detected in basal or DMSO‐incubated NALM‐6 cells. Conversely, NALM‐6 cells treated with DEC demonstrated stable *HOXA9* gene expression. REH and NALM‐6 cells did not express *MEIS1* following DMSO or DEC incubation. *n* = 3, mean ± SD; paired *t* test of Δ*C*_t_ vs *GAPDH* values. (D) Quantitative analysis of protein expression. No MEIS1 protein expression was detected in SEM, and REH cells. *n* = 3, mean ± SD; ratio paired *t* test of absolute GAPDH‐normalized signal intensities. *P < 0.05; **P < 0.01; ***P < 0.001.

We then analyzed the effect of DEC on KMT2A downstream molecules HOXA9 and MEIS1. Focusing on promoter methylation, the hypomethylating substance DEC induced significant promoter demethylation of *HOXA9*, and *MEIS1* in REH and NALM‐6 cells (Fig. 2A,B). The basal promoter methylation in SEM, and RS4;11 cells was very low overall and ranged from 0% to 17% for single CpG islands. No trend toward demethylation was observed after decitabine exposure.

As expected, the decreased promoter methylation in REH, and NALM‐6 cells resulted in elevated gene expression of *HOXA9* (Fig. 2C). This is of special interest as no, and very low basal *HOXA9* gene expression could be detected in NALM‐6 and REH cells, respectively (Fig. S2). In contrast, *MEIS1* transcription, which was basally absent in REH, and NALM‐6 cells, could not be reactivated through DEC incubation. *HOXA9* and *MEIS1* gene expression in SEM, and RS4;11 cells remained constant, matching the methylation analyses. The abundance of the HOXA9 protein was not relevantly altered in any cell line while MEIS1 was decreased in NALM‐6 (Fig. 2D).

Taken together, the initially observed *HOXA9* and *MEIS1* activation was restricted to epigenetic, and gene expression profiles, while translation into HOXA9 and MEIS1 protein upregulation was impaired.

### 3.3 KMT2A translocations are associated with KMT2A pathway gene expression

To further study the importance of basal *KMT2A*, *DNMT1*, *HOXA9*, and *MEIS1* gene expression as well as promoter methylation, we expanded our study cohort and included 12 adult B‐cell precursor ALL primary samples previously amplified in a patient‐derived xenograft model [34]. Since we observed prominent differences between cell lines harboring KMT2A translocations (SEM, RS4;11), and KMT2A wild‐type lines (REH, NALM‐6), we also distinguished between KMT2A‐r, and wild‐type primary samples.

**Fig. 3:**
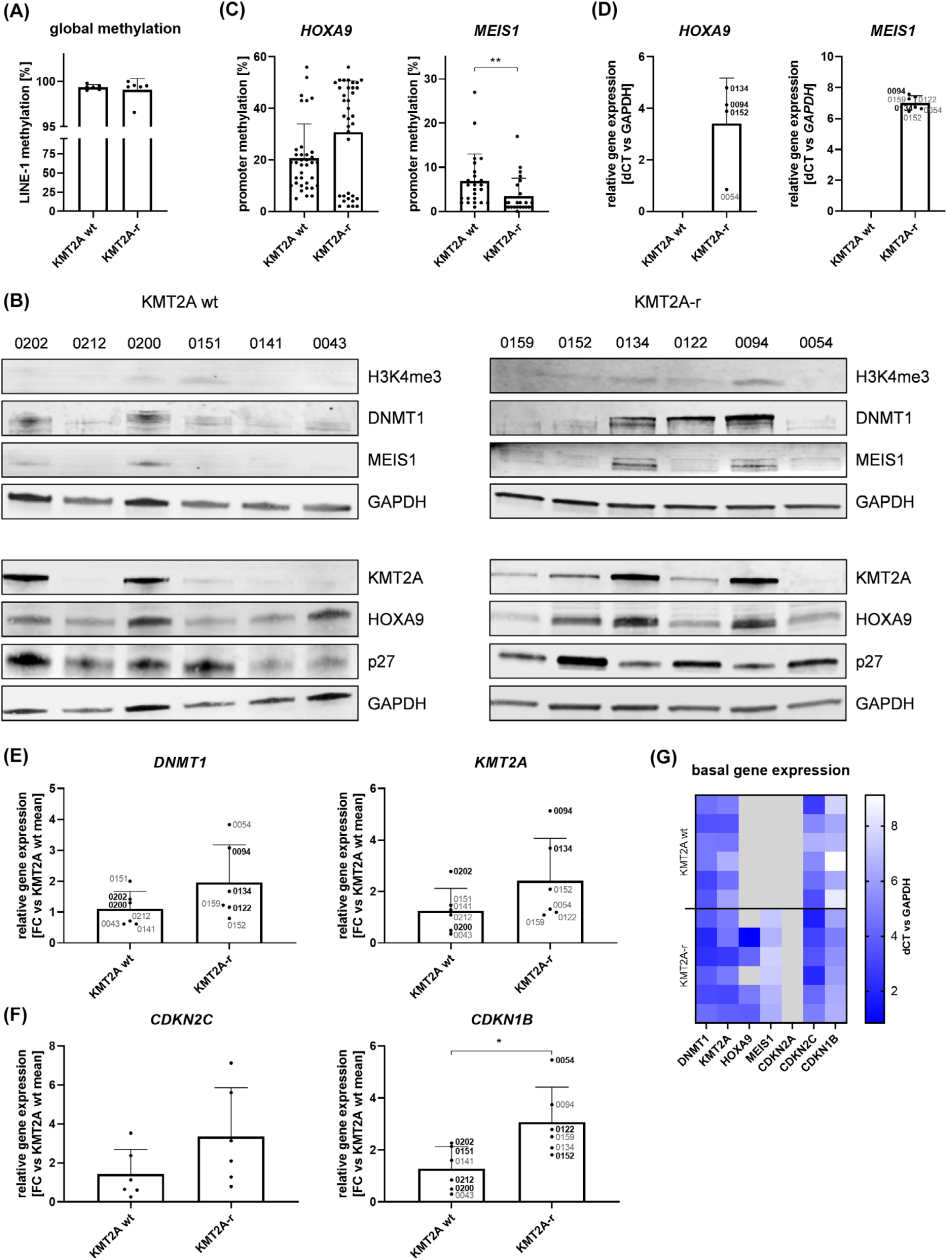
Basal characterization of primary acute B‐lymphoblastic leukemia samples. Blasts were expanded in 12 orthotopic patient‐derived xenograft (PDX) models with wild‐type (wt) KMT2A (*n* = 6) or KMT2A translocation (KMT2A‐r) (*n* = 6), and isolated from spleens. (A) Assessment of global methylation using LINE‐1 methylation‐specific qPCR. Mean ± SD, Mann–Whitney test (not significant). (B) Basal protein expression of histone 3, lysine residue 4 trimethylation (H3K4me3), DNMT1, KMT2A, HOXA9, MEIS1, and p27 in primary samples with KMT2A wt (left panel) or translocation (right panel). GAPDH was used as loading control. *n* = 1. (C) *HOXA9* and *MEIS1* promoter methylation analysis using PyroMark assays. Evaluation of six (*HOXA9*) or four (*MEIS1*) individual CpG islands; each dot represents one CpG island of one sample. Mean ± SD, Mann–Whitney test. (D) Relative *HOXA9*, and *MEIS1* gene expression of KMT2A‐r samples. Demonstration of Δ*C*_t_ vs *GAPDH* values. The primary patient ID is stated in gray alongside the corresponding dot. Bold and black writing indicates high protein expression. No expression was detected in KMT2A wt samples. Mean ± SD. (E) Relative *DNMT1* and *KMT2A* gene expression of KMT2A‐r samples compared to KMT2A wt (fold change, FC). The primary patient ID is stated in gray alongside the corresponding dot. Bold and black writing indicates high protein expression. Mean ± SD, unpaired *t* test (not significant). (F) Relative *CDKN2C* and *CDKN1B* gene expression of KMT2A‐r samples compared to KMT2A wt. The primary patient ID is stated in gray alongside the corresponding dot. Bold and black writing indicates high protein expression. Mean ± SD, unpaired *t* test. (G) Heatmap displaying the basal gene expression in KMT2A wt (upper panel), and rearranged samples (lower panel). Lighter color indicates lower gene expression. Gray fields mark samples with no measurable gene expression. *P < 0.05; **P < 0.01.

No difference in global methylation was observed between the two study groups (Fig. 3A), matching comparable H3K4me3 levels suggesting equal overall transcription susceptibility (Fig. 3B). *MEIS1* promoter methylation was significantly lower in KMT2A‐r samples (Fig. 3C), explaining the high gene expression in this cohort (Fig. 3D). Conversely, *HOXA9* promoter methylation was variable but generally higher in the KMT2A‐r group. Accordingly, the highest methylation values were measured in samples that did not express *HOXA9* at all (#0122, #0159). No *HOXA9* or *MEIS1* transcripts were detected in KMT2A wild‐type samples irrespective of their corresponding promoter methylation profile. *DNMT1* and *KMT2A* gene, and protein expression was slightly higher in KMT2A‐r samples (Fig. 3B,E). Analyzing a broader dataset containing 71 KMT2A‐r, and 102 KMT2A wild‐type samples retrieved from five different patient studies [35, 36, 37, 38, 39] confirmed the results demonstrated in our cohort for *HOXA9*, and *MEIS1* (Figs S3 and S4). In contrast, no difference was observed for *DNMT1*, and *KMT2A* expression, regardless of whether various cytogenetic subgroups or (as in our cohort) only *BCR::ABL1*‐positive samples were used as a control group. Proteomic data of ALL cell lines mirrored this pattern.

To sum up, regarding gene expression patterns especially of KMT2A downstream targets *HOXA9* and *MEIS1* we observed intense differences between KMT2A wild‐type, and rearranged samples.

### 3.4 KMT2A and DNMT1 degradation correlates with DEC biological downstream efficacy

**Fig. 4:**
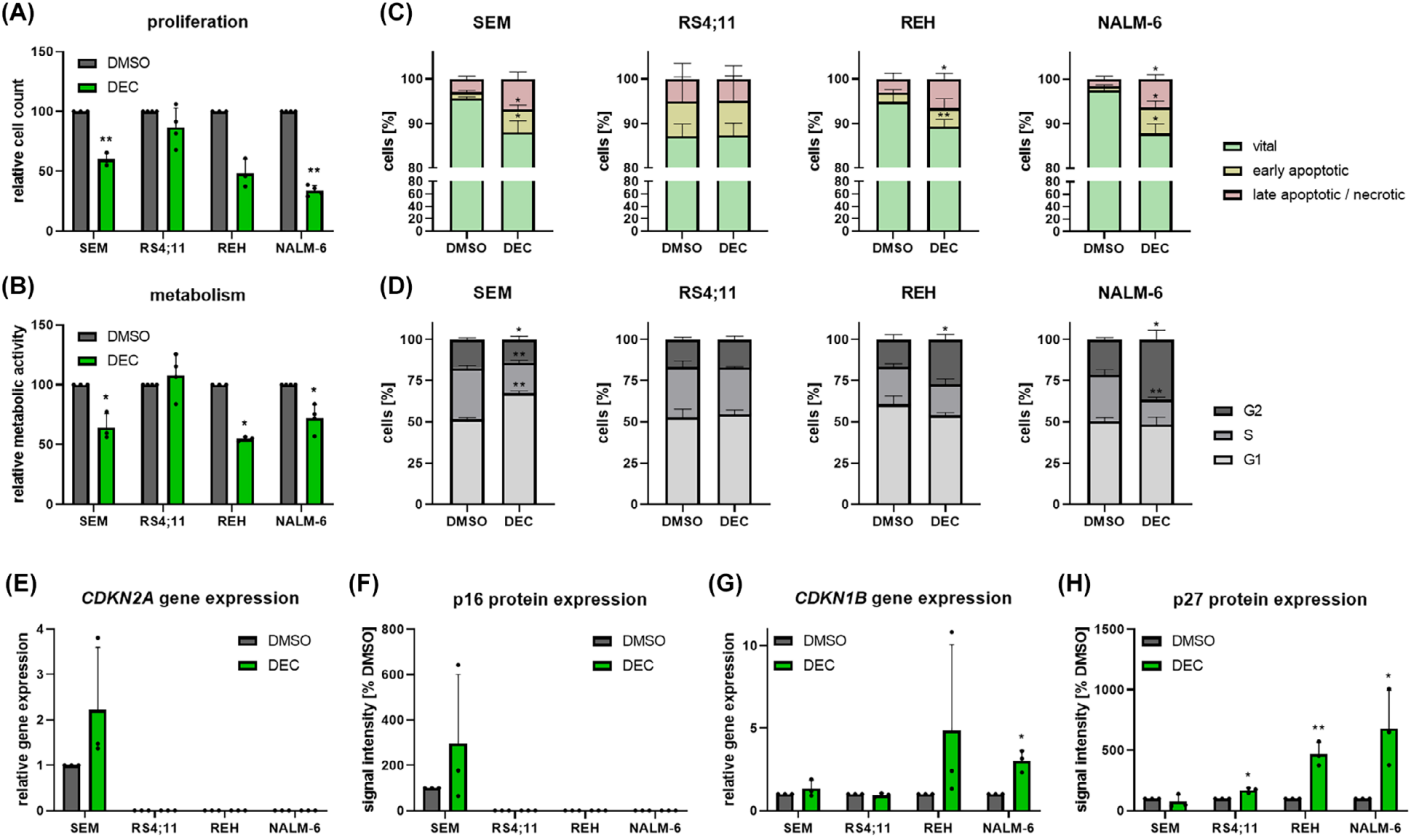
Influence of Decitabine (DEC) on biological processes in acute B‐lymphoblastic leukemia cell lines SEM, RS4;11, REH, and NALM‐6. (A) To assess proliferation, cells were incubated with 100 nm DEC or control (DMSO) for 72 h before trypan blue staining. SEM, REH: *n* = 3; RS4;11, NALM‐6: *n* = 4, mean ± SD; paired *t* test of absolute cell counts. (B) WST‐1 assay for the analysis of cellular metabolism following 72 h incubation with 100 nm DEC. SEM, REH: *n* = 3; RS4;11, NALM‐6: *n* = 4, mean ± SD; paired *t* test of absolute signal intensities. (C) Analysis of apoptotic processes after 72 h of 100 nm DEC using Annexin V/propidium iodide (PI) staining, and flow cytometry. *n* = 3, mean ± SD; paired *t* test. (D) Cell cycle phases (G2/M, S, G0/G1) were determined by PI staining, and subsequent flow cytometry after 72 h DEC incubation (100 nm). *n* = 3, mean ± SD; paired *t* test. (E, G) Probe‐based gene expression analysis of *CDKN2A* (E), and *CDKN1B* (G) following 72 h 1 μm DEC incubation. *n* = 3, mean ± SD; paired *t* test of Δ*C*_t_ vs. *GAPDH* values. (F, H) Quantitative analysis of p16 (F), and p27 (H) protein expression after 72 h incubation with 1 μm DEC. *n* = 3, mean ± SD; ratio paired *t* test of absolute GAPDH‐normalized signal intensities. *P < 0.05; **P < 0.01.

To further investigate the biological impact of DEC incubation on the KMT2A‐r, and wild‐type cell lines, we first evaluated the influence on proliferation and metabolic activity. Except for RS4;11 cells, all cell lines demonstrated significantly reduced cell counts, and metabolism (Fig. 4A,B). Further, siRNA‐mediated knockdown of both KMT2A or DNMT1 resulted in comparable results in SEM, and NALM‐6 cells (Fig. S5A–C). This matches our previous findings, suggesting that global demethylation, overall transcriptional activation as well as degradation of KMT2A, and DNMT1 is responsible for the biological downstream efficacy of DEC in SEM, and RS4;11 cells. Dual KMT2A or DNMT1 targeting using DEC in combination with siRNA‐mediated knockdown of either gene did not increase the anti‐leukemic effects induced by DEC alone (Fig. S5D).

Finally, while again no effect was detected in RS4;11 cells, DEC also significantly induced apoptosis and cell cycle changes in SEM, REH, and NALM‐6 cells (Fig. 4C,D). Interestingly, a G1 phase arrest was observed in KMT2A‐r cell line SEM, whereas KMT2A wild‐type cell lines REH, and NALM‐6 were locked in the G2 phase. This interesting finding prompted us to investigate if the differential cell cycle regulation is related to KMT2A translocations in general. We therefore analyzed the gene and protein expression of three cell cycle regulators in all four cell lines as well as 12 primary B‐ALL samples. Basal expression of *CDKN2A*, translating into the p16^*INK4A*^ protein, was only detected in SEM cells (Fig. S2, Fig. 4E). *CDKN2C* (p18^*INK4C*^), and *CDKN1B* (p27^*Kip1*^) were present in all samples, although gene expression rates were significantly higher in KMT2A‐r cells (Fig. S2, Fig. 3B,F,G).

Incubation with DEC resulted in an increase in *CDKN2A*/p16^*INK4A*^ expression in KMT2A‐r SEM cells (Fig. 4E,F), probably contributing to the observed G1 arrest [43]. In contrast, *CDKN1B*/p27^*Kip1*^ was significantly upregulated in KMT2A wild‐type cell lines REH, and NALM‐6, with DEC elevating the protein abundance to over 500% compared to control cells (Fig. 4G,H). p27^*Kip1*^ is known to interfere with the G2/M cell cycle checkpoint in cancer [44], explaining the G2 phase arrest seen in KMT2A wild‐type cell lines REH and NALM‐6. The gene expression of *CDKN2C* remained constant in RS4;11 cells, and was mildly increased in all other cell lines irrespective of KMT2A status (Fig. S6), probably attributing to a general and not cell cycle‐ or translocation‐specific DEC‐mediated effect.

In summary, we observed that cell lines with good biological response also experienced DEC‐attributed KMT2A degradation. This effect seems to be evoked via cell cycle arrest, but the type of modulation is variable, and might differ between KMT2A‐r and wild‐type cells.

### 3.5 DEC influences methyltransferase activity and expression *in vivo*

**Fig. 5:**
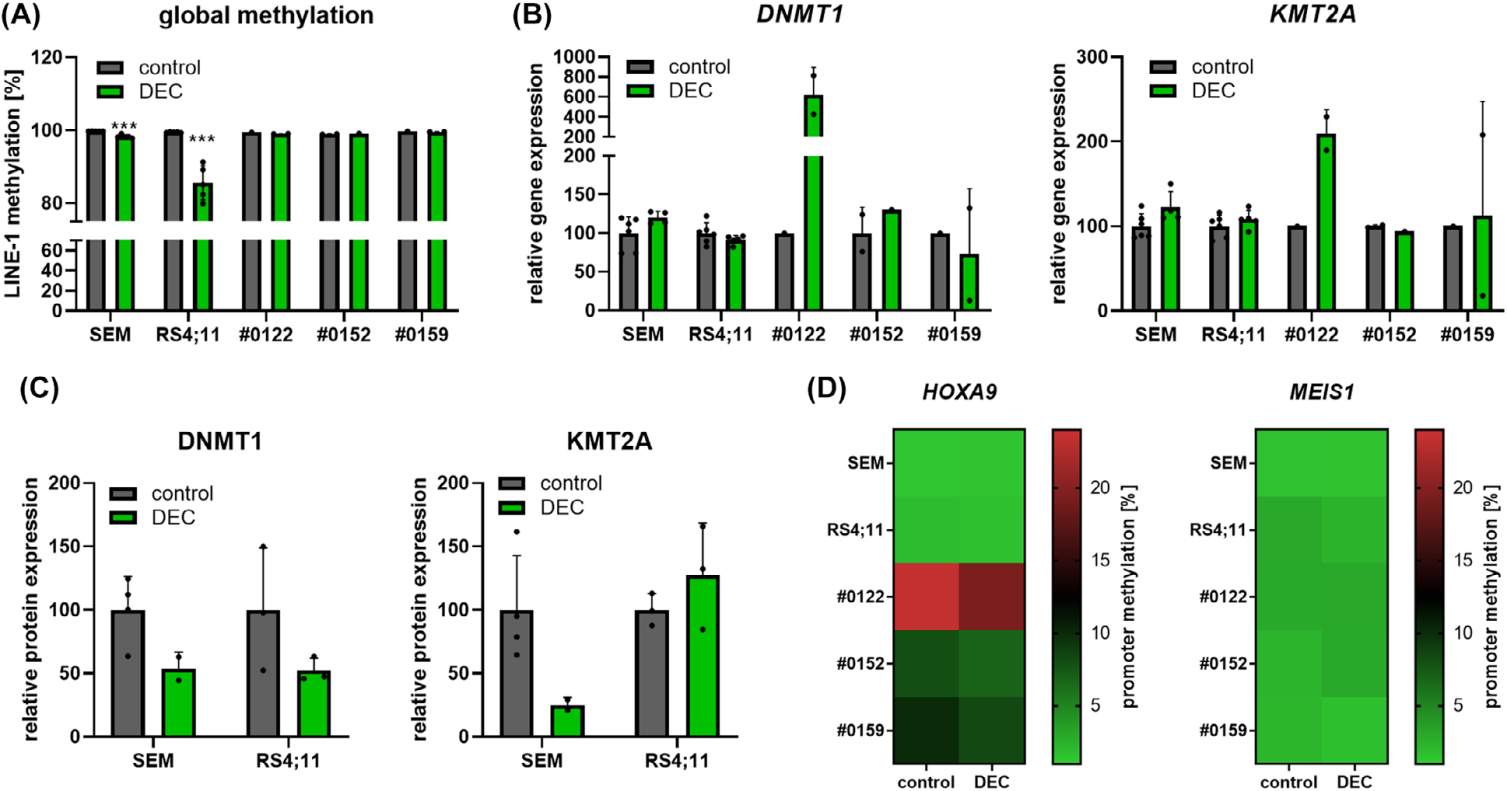
*In vivo* response to Decitabine (DEC) in KMT2A‐rearranged cell lines and primary samples. Four to six (cell line models) or one to two mice (patient‐derived xenograft models) per study group were treated for four consecutive days starting 1 week after tumor cell injection. The exact number of animals analyzed per study group differs between DNA‐, RNA‐, and protein‐based methods due to different sample availability. The number of samples is indicated by the number of dots in the respective figure, with each dot representing an individual animal. Blasts were isolated, and analyzed after 30 days. Each dot symbol represents an individual animal. (A) Assessment of global methylation using *LINE‐1* methylation‐specific qPCR. Mean ± SD, unpaired *t* test for cohorts with at least three individual samples in both control and treatment group. (B) Probe‐based gene expression analysis of *DNMT1* and *KMT2A*. Mean ± SD; unpaired *t* test of Δ*C*_t_ vs *GAPDH* values relative to the mean of all control animals of the respective model for cohorts with at least three individual samples in both, control, and treatment group (not significant). (C) Quantitative analysis of DNMT1, and KMT2A protein expression. Mean ± SD; unpaired *t* test of absolute GAPDH‐normalized signal intensities (not significant). (D) Analysis of the promoter methylation using PyroMark assays. Six (*HOXA9*) or four (*MEIS1*) CpG islands were evaluated. Heatmap displaying the overall promoter methylation. ***P < 0.001.

We next aimed to elucidate the direct effects of DEC on DNMT1, and KMT2A as well as on downstream signaling molecules in an *in vivo* setting. As KMT2A wildtype cell lines, and primary samples do not express *HOXA9* and *MEIS1*, we restricted those follow‐up analyses to two KMT2A‐r cell lines (SEM, RS4;11) and three KMT2A‐r patient‐derived xenograft models (#0122, #0152, #0159). DEC was tolerated well by all mice, and induced a significant delay in tumor cell proliferation, as previously published [16]. Global LINE‐1 methylation levels were very high in all models. In contrast to *in vitro* studies, DEC induced demethylation mainly in RS4;11‐derived xenografts while patterns remained unaffected in primary samples (Fig. 5A).

Gene expression profiles of methyltransferases *DNMT1*, and *KMT2A* remained stable in SEM‐ and RS4;11‐derived xenograft models as well as two out of three primary samples, matching the results from the previous experiments (Fig. 5B). Mice engrafted with cells of patient #0122 experienced a strong increase of both *DNMT1*, and *KMT2A* gene expression. Strikingly, and in line with our *in vitro* observations, DEC treatment resulted in not only DNMT1 degradation but also reduced KMT2A protein expression levels in SEM‐derived mice (Fig. 5C).

Further investigating downstream processes, we first evaluated *HOXA9*, and *MEIS1* promoter methylation (Fig. 5D, Fig. S7A). The *MEIS1* promoter was unmethylated in all samples and models, thus showing no demethylating effects following DEC treatment. Basal *HOXA9* promoter methylation was also absent in cell line‐derived models. However, primary samples had slightly higher methylation values, and promoter methylation mildly decreased after DEC treatment. Interestingly, mice engrafted with cells derived from patient #0122, who experienced strong upregulation of *DNMT1*, and *KMT2A* gene expression after therapy, displayed the highest *HOXA9* promoter methylation values. In line, no *HOXA9* gene expression was detected in this model (Fig. S7B).

All other animal models showed stable *HOXA9*, and *MEIS1* transcription. Treatment with DEC resulted in a mild elevation of gene expression values (Fig. S7B), probably attributed to overall demethylation. Matching the *in vitro* results, HOXA9 and MEIS1 protein expression profiles remained largely unaffected following DEC treatment (Fig. S7C). This data demonstrates that the herein identified DEC‐associated KMT2A degradation is also present in an *in vivo* setting.

### 3.6 Dual targeting of KMT2A synergistically increases anti‐leukemic effects

Finally, we investigated if dual KMT2A targeting by DEC in combination with menin inhibitor revumenib (REV) could potentiate the observed effects. Menin is a critical co‐factor of KMT2A, and menin inhibitors are a novel and promising treatment option in KMT2A‐r leukemias [45]. As menin inhibitors are only considered for KMT2A‐r patients, further studies were extended to KMT2A translocation harboring AML cell lines MOLM13, and MV4;11. Initial dose–response profiling demonstrated a concentration‐dependent reduction in proliferation, metabolic activity with IC50 concentrations in nanomolar ranges, and tumor cell viability in SEM and MV4;11 cells, which were therefore used for subsequent combination studies (Fig. S8). On the other hand, primary REV resistance was discovered in RS4;11, and MOLM13 cells as well as KMT2A wt lines REH and NALM‐6.

**Fig. 6:**
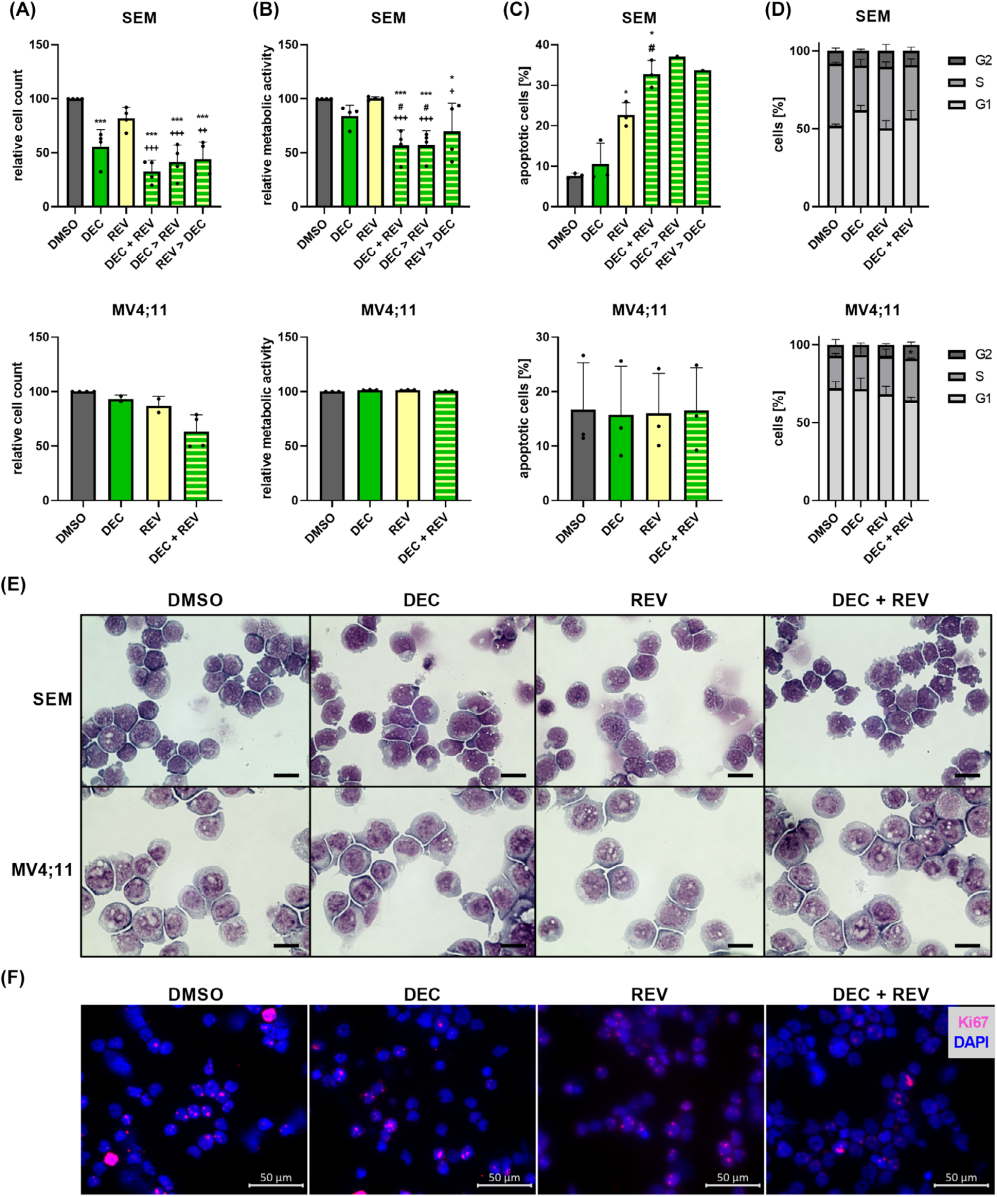
Biological efficacy of combined Decitabine (DEC, 100 nm), and Revumenib (REV, 10 nm) incubation in KMT2A‐rearranged cell lines SEM and MV4;11. (A–D) Substances were applied either alone, simultaneously (DEC + REV), or the second inhibitor was added 24 h after the first drug (DEC > REV or REV > DEC), and readout parameters proliferation (A; trypan blue staining), metabolism (B; WST‐1 assay), apoptosis induction (C; annexin V/propidium iodide (PI) staining and flow cytometry), and cell cycle phases (D; PI staining and flow cytometry) were determined 72 h after the first inhibitor was applied. SEM (apoptosis sequential) *n* = 1; MV4;11 (proliferation DEC, REV) *n* = 2; SEM (apoptosis control, DEC, REV, simultaneous, cell cycle), MV4;11 (metabolism, apoptosis, cell cycle) *n* = 3; SEM (proliferation, metabolism), MV4;11 (proliferation control, combination) *n* = 4, mean ± SD; repeated measures one‐way ANOVA, and post‐hoc Tukey's multiple comparisons test for comparisons with at least three biological replicates in each group. Significance tested vs. control (DMSO) (*), DEC mono application (#), or REV mono application (+). (E) Assessment of cell morphology after 72 h incubation using Pappenheim staining. Representative images of three independent biological replicates at 100× magnification; 20 μm scale bar. (F) Analysis of proliferation marker Ki67 (red), and nuclei (DAPI, blue) following 72 h incubation by immunofluorescence staining. Representative images of three independent biological replicates. *^/#/+^ *P* < 0.05; ^++^ *P* < 0.01; ***^/+++^ *P* < 0.001.

In contrast, combined application of DEC and REV using IC20/30 concentrations revealed significantly, and synergistically reduced tumor cell proliferation, metabolism, and vitality in SEM cells (Fig. 6, Table S4). Sequential incubation resulted in similar effects. Apoptosis was accompanied by a reduction of cell size, membrane blebbing, and loss of Ki67 staining, especially in the combination group. At the same time, little to no changes were observed in the cell line MV4;11.

As the combined application of DEC and REV has not been investigated either preclinically or clinically before and because it is known that both DEC and REV induce neutropenia, and anemia, we also assayed the effect on healthy blood cells. As expected, both drugs reduced PBMC viability (Fig. S8D) but the combination did not increase cytotoxicity. No hemolysis could be detected following either mono or combined inhibitor application (Fig. S8E).

Investigating potential modes of synergistic action, we found that REV did not extend DEC‐induced global demethylation (Fig. S9A). Further, REV alone or in combination with DEC did not result in relevant or synergistic modulation of *KMT2A*, *DNMT1*, *HOXA9*, or *MEIS1* expression (Fig. S9B). Expression of the *KMT2A::AFF1* fusion gene also remained mainly stable, although a trend toward decreased transcriptional activity was present following both substances as well as the combination using low nanomolar concentrations in SEM cells (Fig. S9C). Here, DEC achieved an inhibition comparable to the known KMT2A fusion inhibitor REV, underlining the potential of DEC as anti‐KMT2A agent, and therefore mediator of downstream leukemic pathways.

In summary, simultaneous or sequential DEC, and REV application acts synergistically in SEM cells, demonstrating in principle the anti‐leukemic capability of this combination. However, the mode of action as well as suitable biomarkers to predict the response to this treatment must be identified in future studies.

## 4 Discussion

The hypomethylating agent DEC is successfully used in the clinical setting. However, to date only elderly patients with AML or myelodysplastic syndromes who are unfit for intensive chemotherapy routinely profit from DEC [11, 12]. Although the clinical benefit in those cohorts is high, and despite promising preclinical studies [16, 46], the availability of DEC in other hematological malignancies with adverse prognoses is unsatisfactory. Also, the substance's mode of action is still not completely understood, with novel mechanisms being discovered persistently [47, 48, 49]. However, those studies are usually restricted to AML samples, while other hematological subtypes remain uninvestigated. Preclinical analyses [16, 46] and occasional case reports [13, 14] demonstrate that adult B‐ALLs, featuring high relapse rates, and grim prognoses, might benefit from DEC treatment. We therefore aimed to elucidate the effect of DEC in this cohort in more detail.

In infant ALL, it was shown that DEC was able to extend the survival in xenograft models of KMT2A‐r samples, and induces apoptosis in KMT2A‐r cell lines [46, 50]. Both studies also demonstrated depletion of DNMT1, matching our findings in both KMT2A‐r, and wild‐type cell lines. Using guadecitabine, a second generation DNMT1 inhibitor, a different group recently further demonstrated upregulation of *KMT2A* gene expression [51]. We observed similar effects in our samples using high, and low DEC concentrations, attributing to the fact that different mechanisms are activated dependent on the DEC dosage [52]. Intriguingly, all approaches resulted in a strong reduction of KMT2A protein levels, and this degradation only occurred following DEC incubation and not in response to other cell death‐inducing agents. This observation rules out the option of unspecific cytotoxic or cell stress effects as a source of KMT2A degradation, as those were present in all samples. This finding is of special relevance because KMT2A can also act as transcription factor, suggesting that overexpression results in upregulation of KMT2A target genes like *HOXA9* [53]. Increased KMT2A expression was shown in AML, and CMML patients compared to controls [54, 55], and other groups demonstrated that high KMT2A levels were associated with tumor cell proliferation and migration in solid tumors [56, 57, 58, 59]. Also, high KMT2A expression predicted poor survival in hepatocellular carcinoma, and melanoma patients [57, 60], demonstrating the potential clinical benefit of KMT2A degradation in both KMT2A‐r and wild‐type patients. Still, the data suggest that the underlying mechanisms of DEC efficacy might differ between both populations, and several mechanisms besides reduced KMT2A activity contribute to the observed anti‐tumorigenic effects.

During ALL therapy, resistance frequently occurs, and combination strategies as well as dual targeting approaches are used to address this problem. In this study, we performed two different combinatorial experiments to target KMT2A for increased efficacy. Parallel DEC application, and *KMT2A* gene knockdown did not increase the anti‐leukemic effects of DEC monotherapy. However, these experimental data might be hampered by siRNA knockdown efficacy, and longevity. A further limitation is the lack of parallel assessment of protein expression.

In contrast, combined DEC, and REV incubation resulted in significantly and synergistically reduced proliferation and metabolic activity in SEM cells, suggesting that this previously uninvestigated dual KMT2A intervention indeed possesses anti‐leukemic potential. Importantly, synergistic effects were only observed in SEM cells but not in the cell line MV4;11 which was also susceptible to both DEC and REV but demonstrated an additive or antagonistic response. Gene expression patterns differed between the two cell lines, with reductions in *DNMT1*, *KMT2A*, *HOXA9*, and *MEIS1* in SEM cells while those markers were rather upregulated in the MV4;11 cell line. This trend was also observed for the *KMT2A::AFF1* fusion gene, although changes were small and not statistically significant. However, as only low nanomolar concentrations were used, only small changes were to be expected. Further, the fact that DEC induced similar or higher *KMT2A::AFF1* as well as *HOXA9* downregulation compared to the KMT2A fusion inhibitor REV strongly supports the hypothesis of KMT2A degradation‐mediated anti‐leukemic effects in this setting. To expand this data, and knowledge about this mechanism, future experiments should investigate the effect of DEC on KMT2A fusion molecules on protein level.

It is therefore important to further investigate the mechanisms behind the synergistic anti‐leukemic capability, and identify suitable biomarkers of response, especially in the light of potential risks of DEC or REV treatment. As known from clinical observations, DEC [11, 61, 62] and REV [45] frequently induce neutropenia, matching our experimental data of reduced PBMC viability following both DEC or REV incubation. Still, combined application did not result in increased PBMC impairment, suggesting that the dual application might have a toxicity profile compared to mono‐treatment.

Due to the high structural similarity between DNMT1, and KMT2A DNA‐binding CXXC domains, we hypothesize that DEC binds not only DNMT1 but also KMT2A. Once incorporated into the DNA within CpG islands during replication, DNMT1 inhibitors like DEC or GSK‐3484862 bind the methyltransferase mainly via the carbon‐5, and carbon‐6 atoms of the pyrimidine structure [63]. Due to the modification in the carbon‐5 position of decitabine, this bond cannot be released, and results in a covalent trapping of DNMT1, targeting the structure for proteasomal degradation [10, 64]. In our data, all cell lines with reduced DNMT1 protein levels also featured reduced global methylation, and increased euchromatin availability following DEC incubation. Strikingly, those cells also had diminished KMT2A protein expression. Biological efficacy, including decreased cell proliferation, metabolism, and vitality, was again restricted to those cells that experienced KMT2A degradation. Transcriptional silencing of KMT2A also resulted in reduced tumor cell proliferation, matching existing data in CMML [55]. Those observations suggest a role for KMT2A degradation in DEC functionality; however, it is likely that several different modes of action simultaneously contribute to the anti‐leukemic effects. Finally, an *in vivo* approach confirmed our previous findings, and demonstrated reduced DNMT1 and KMT2A protein expression following DEC therapy in SEM‐derived xenografts. In contrast, mice injected with RS4;11 cells showed stable KMT2A expression, matching the *in vitro* results. The lack of downstream regulation of *HOXA9*, and *MEIS1* in the *in vivo* setting might be explained by the long time between the final DEC dose (day 10) and the tissue collection (day 31).

As the DNA‐binding CXXC domain of KMT2A also recognizes unmethylated CpG islands, and specifically interacts with the same cytosine atoms as DNMT1, these results suggest that the interaction of the KMT2A CXXC domain, and incorporated DEC results in KMT2A inactivation and degradation via comparable mechanisms. Therefore, our data indicate that, besides the known mechanisms of TSG demethylation, and DNMT1 degradation, DEC also depletes KMT2A as a novel and previously undescribed mode of action. This is likely evoked by direct binding of DEC to KMT2A, although x‐ray diffraction studies or other experiments to decipher the chemical interaction remains to be conducted to ultimately prove which atoms are involved.

To validate our finding and investigate downstream effects, we further analyzed the KMT2A targets HOXA9, and MEIS1. As expected, DEC demethylated the promoters of both transcription factors, and thus increased gene expression in REH and NALM‐6 cells. This process was likely mediated via the already known modes of action: inhibition of DNMT1 and inability to be methylated itself, with subsequent loss of CpG methylation. In contrast, cell lines SEM and RS4;11 which presented a commonly observed pattern in KMT2A‐r cells with low promoter methylation and thus very high gene expression, were not further demethylated. However, in contrast to heterogeneous gene expression data, protein levels were stable, or decreased across all four cell lines *in vitro* and *in vivo*. This might be due to posttranslational modification. On the other hand, HOXA9 activity is dependent on KMT2A binding to the *HOXA9* locus [65, 66, 67], and with KMT2A being degraded following DEC incubation, this activation would be hampered.

KMT2A translocations also play a role in *HOXA9* expression [68]: in our data set, the gene expression profile of adult KMT2A‐r B‐ALL primary samples is distinct from KMT2A wild‐type patients. Basal *HOXA9*, and *MEIS1* expression is restricted to patients with rearrangements, matching existing literature [69] and undermining the potential of DEC for the treatment of this patient cohort.

Possibly regulated by different basal transcription factor expression, we also detected differences in the biological response of KMT2A‐r (SEM), and wild‐type (REH, NALM‐6) cell lines to DEC. KMT2A is a central regulator of the S phase entry, with KMT2A translocations leading to overruling of the checkpoint and thus increased cell division [70]. Demonstrating that functional KMT2A is necessary for S phase entry, Liu et al. [71] showed that cells lacking KMT2A remain in the G1 phase. This is in line with our data on KMT2A‐r SEM cells: DEC‐induced KMT2A degradation resulted in a significant G1 phase arrest. As a possible mode of action, this was accompanied by p16 upregulation, a cyclin dependent kinase inhibitor targeting CDK4/6 and thus preventing G1‐S transit [72]. In contrast, KMT2A wild‐type cell lines REH, and NALM‐6 experienced a strong p27 upregulation and subsequent G2 phase arrest, likely mediated via p27‐induced CDK1/2 inhibition [44, 72]. Although our observation of different cell cycle modulatory effects in cells with and without KMT2A rearrangements fit in well with the existing scientific literature, definite conclusions cannot be made due to the limited amount of samples investigated in each cohort. It is, however, known that KMT2A actively regulates p18 and p27 cell cycle regulators, thus supporting our hypothesis [27, 73].

## 5 Conclusions

Overall, these data together with the convincing clinical efficacy underline the capacity of DEC as potent agent in acute leukemia therapy, and further elucidate a novel mode of action. Probably due to structural similarity, DEC not only degrades DNMT1 but also KMT2A, a leukemic driver frequently affected by chromosomal translocations. Demonstrating broad response independent of KMT2A rearrangement status, we suggest that DEC should be considered as an additional therapeutic option in adult acute lymphoblastic leukemia.

## Conflict of interest

The authors declare no conflict of interest.

## Author contributions

ARi contributed to conceptualization, writing—original draft, and project administration; LBr, SL, CR, MH, HME, and ARi contributed to methodology; LBr, SL, and ARi contributed to validation; LBr, SL, MH, HME, and ARi contributed to formal analysis; LBr, LBe, SL, ARu, CR, MH, AS, GK, HME, and ARi contributed to investigation; MH, HME, CJ, and ARi contributed to resources; LBr, SL, HME, CJ, and ARi contributed to writing—review and editing; LBr and ARi contributed to visualization; ARi, HME, and CJ contributed to supervision; HME, CJ, and ARi contributed to funding acquisition.

### Peer review

The peer review history for this article is available at https://www.webofscience.com/api/gateway/wos/peer‐review/10.1002/1878‐0261.13792.

## Supporting information


**Fig. S1.** Concentration‐dependent effects of DEC on gene, and KMT2A protein expression in SEM cells.
**Fig. S2.** Basal gene expression in cell lines (SEM, RS4;11, REH, NALM‐6), primary samples (four‐digit numbers), and five healthy donor B‐cells.
**Fig. S3.** Gene and protein expression of DNMT1, KMT2A, HOXA9, and MEIS1.
**Fig. S4.** Gene and protein expression of DNMT1, KMT2A, HOXA9, and MEIS1.
**Fig. S5.** Influence of siRNA‐mediated transcriptional silencing of KMT2A and DNMT1 on cell proliferation, and decitabine response in SEM and NALM‐6 cells.
**Fig. S6.** Probe‐based gene expression analysis of CDKN2C following 72 h 1 μm DEC incubation.
**Fig. S7.** DEC‐mediated effects on HOXA9, and MEIS1 in xenograft model systems.
**Fig. S8.** Concentration‐dependent effects of menin inhibitor revumenib (REV) on acute leukemia cell lines.
**Fig. S9.** Effects of simultaneous 72 h DEC (100 nm), and REV (10 nm) incubation on methyltransferase‐mediated signaling pathways.

**Table S1.** Western blot antibodies.

**Table S2.** Primers for gene expression analyses.

**Table S3.** Patient characteristics.

**Table S4.** Bliss synergy values for combined decitabine (DEC), and revumenib (REV) incubation.

## Data Availability

All raw and processed data are available from the corresponding author upon reasonable request.
