# Supplementary material for: KMT2A degradation is observed in decitabine‐responsive acute lymphoblastic leukemia cells
Source: Mol Oncol. 2025 Jan 4;19(5):1404–21. doi: 10.1002/1878-0261.13792 (PMC12077275; doi:10.1002/1878-0261.13792)
Supplement: Supplementary file 1 — Fig. S1. Concentration‐dependent effects of DEC on gene, and KMT2A protein expression in SEM cells. Fig. S2. Basal gene expression in cell lines (SEM, RS4;11, REH, NALM‐6), primary samples (four‐digit numbers), and five healthy donor B‐cells. Fig. S3. Gene and protein expression of DNMT1, KMT2A, HOXA9, and MEIS1. Fig. S4. Gene and protein expression of DNMT1, KMT2A, HOXA9, and MEIS1. Fig. S5. Influence of siRNA‐mediated transcriptional silencing of KMT2A and DNMT1 on cell proliferation, and decitabine response in SEM and NALM‐6 cells. Fig. S6. Probe‐based gene expression analysis of CDKN2C following 72 h 1 μm DEC incubation. Fig. S7. DEC‐mediated effects on HOXA9, and MEIS1 in xenograft model systems. Fig. S8. Concentration‐dependent effects of menin inhibitor revumenib (REV) on acute leukemia cell lines. Fig. S9. Effects of simultaneous 72 h DEC (100 nm), and REV (10 nm) incubation on methyltransferase‐mediated signaling pathways. [file MOL2-19-1404-s001.zip › FiguresS1-S9_Legends.docx]

**Legends for Supplementary Figures**

**Figure S1: Concentration-dependent effects of Decitabine (DEC) on gene and KMT2A protein expression in SEM cells. A**Cells were incubated with 0.5 µM or 1.0 µM DEC for 72 h before probe-based gene expression analysis. n=3, mean ± SD. **B**Detection of KMT2A protein level using intracellular flow cytometry of SEM cells incubated with increasing concentrations of DEC for 72 h. n=3, mean ± SD. **C**KMT2A protein expression in response to venetoclax (VEN), silmitasertib (CX4945) and revumenib (REV). n=1.

**Figure S2: Basal gene expression in cell lines (SEM, RS4;11, REH, NALM-6), primary samples (four-digit numbers) and five healthy donor B-cells.** Heatmap displaying the basal gene expression in KMT2A-rearranged (KMT2A-r) samples (upper panel), KMT2A-wild type (wt) samples (middle panel) and healthy control samples (lower panel). Lighter color indicates lower gene expression. Grey fields mark samples with no measureable gene expression.

**Figure S3: Gene and protein expression of DNMT1, KMT2A, HOXA9 and MEIS1. A** Microarray data of acute lymphoblastic leukemia (ALL) patients were retrieved from the GEO platform (accession GSE66006) and analyzed using GEO2R to depict gene expression in KMT2A wild type (left, green) and KMT2A-r samples (right, purple). Each line indicates an individual patient sample. **B** Proteomics data of 51 ALL cell lines were analyzed using the FORALL resource to demonstrate protein expression in KMT2Ar (left, red) and KMT2A wild type (right, blue) samples.

**Figure S4: Gene and protein expression of DNMT1, KMT2A, HOXA9 and MEIS1. A** Microarray data of acute lymphoblastic leukemia (ALL) patients were retrieved from the GEO platform (accession GSE66006) and analyzed using GEO2R to depict gene expression in BCR::ABL1 positive (left, green) and KMT2A-r samples (right, purple). Each line indicates an individual patient sample. **B** Proteomics data of ALL cell lines were analyzed using the FORALL resource to demonstrate protein expression in KMT2Ar (left, red; n=4 cell lines with replicates) and BCR::ABL1 positive (right, blue; n=2 cell lines and replicates) samples.

**Figure S5: Influence of siRNA-mediated transcriptional silencing of KMT2A and DNMT1 on cell proliferation and decitabine (DEC) response in SEM and NALM-6 cells. A** Probe-based gene expression analysis of KMT2A and DNMT1 in siRNA-electroporated cells relative to non-coding control siRNA (siNC). n=6-8, mean ± SD. **B** Trypan blue staining and subsequent cell counting for analysis of proliferation following siRNA electroporation. n=2-4, mean ± SD relative to siNC; repeated measures one-way ANOVA with post-hoc Tukey’s multiple comparisons test. **C** WST1 assay for the analysis of cellular metabolism following siRNA electroporation. n=2-4, mean ± SD relative to siNC; repeated measures one-way ANOVA with post-hoc Tukey’s multiple comparisons test. **D** Assessment of proliferation and metabolic activity after 72 h DEC incubation in cells electroporated with siKMT2A, siDNMT1 or siNC. n=2-4, mean ± SD relative to siNC; repeated measures one-way ANOVA. * p < 0.05; ** p < 0.01.

**Figure S6:** Probe-based gene expression analysis of *CDKN2C* following 72 h 1 µM decitabine (DEC) incubation. n=3, mean ± SD; paired t test of ΔCt vs GAPDH values. ** p < 0.01.

**Figure S7: Decitabine (DEC)-mediated effects on *HOXA9* and *MEIS1* in xenograft model systems.** Four to six (cell line models) or one to two mice (PDX models) per study group were treated for four consecutive days starting one week after tumor cell injection. Blasts were isolated and analyzed after thirty days. Each dot symbol represents an individual animal. **A** Analysis of the promoter methylation using PyroMark assays. Six (*HOXA9*) or four (*MEIS1*) CpG islands were evaluated and the absolute methylation is displayed. Unpaired t test. **B** Probe-based *HOXA9* and *MEIS1* gene expression analysis. Mean ± SD; unpaired t test of ΔCt vs *GAPDH* values relative to the mean of all control animals of the respective model. **C** Quantitative analysis of HOXA9 protein expression. No MEIS1 protein expression was detected in SEM- and RS4;11-derived xenograft models. Mean ± SD; unpaired t test of absolute GAPDH-normalized signal intensities relative to the mean of all control animals of the respective model. * p < 0.05.

**Figure S8: Concentration-dependent effects of menin inhibitor revumenib (REV) on acute leukemia cell lines. A,B** Determination of IC50 concentrations using trypan blue staining and subsequent cell counting for proliferation assessment (**A**) or WST1 assay for metabolic activity (**B**). n=2-3, calculated IC50 values are given in brackets behind the respecitive cell lines. **C** Analysis of apoptotic cell frequency via annexin VFITC and PI staining and subsequent flow cytometry. Early (annexin VFITC+/PI-) and late apoptotic (annexin VFITC+/PI+) cells are summarized. n=2-3, mean ± SD; repeated measures one-way ANOVA with post-hoc Tukey’s multiple comparisons test. No statistical testing was performed for MV4;11 due to insufficient biological replicates (n=2).

**Figure S9: Effects of simultaneous 72 h decitabine (DEC, 100 nM) and revumenib (REV, 10 nM) incubation on methyltransferase-mediated signaling pathways. A** Assessment of global methylation using LINE1 methylation-specific qPCR. n=3; mean ± SD, repeated measures one-way ANOVA vs DMSO control and post-hoc Tukey’s multiple comparisons test. **B** Probe-based gene expression analysis of KMT2A, DNMT1, HOXA9 and MEIS1. n=3; mean ± SD; repeated measures one-way ANOVA vs DMSO control and post-hoc Tukey’s multiple comparisons test of GAPDH-normalized fold change values. * p < 0.05. **C** Probe-based analysis of the KMT2A::AFF1 fusion gene. n=3; mean ± SD; repeated measures one-way ANOVA vs DMSO control and post-hoc Tukey’s multiple comparisons test of GAPDH-normalized fold change values.
