# Supplementary figures and images for: KMT2A degradation is observed in decitabine‐responsive acute lymphoblastic leukemia cells
Source: Mol Oncol. 2025 Jan 4;19(5):1404–21. doi: 10.1002/1878-0261.13792 (PMC12077275; doi:10.1002/1878-0261.13792)

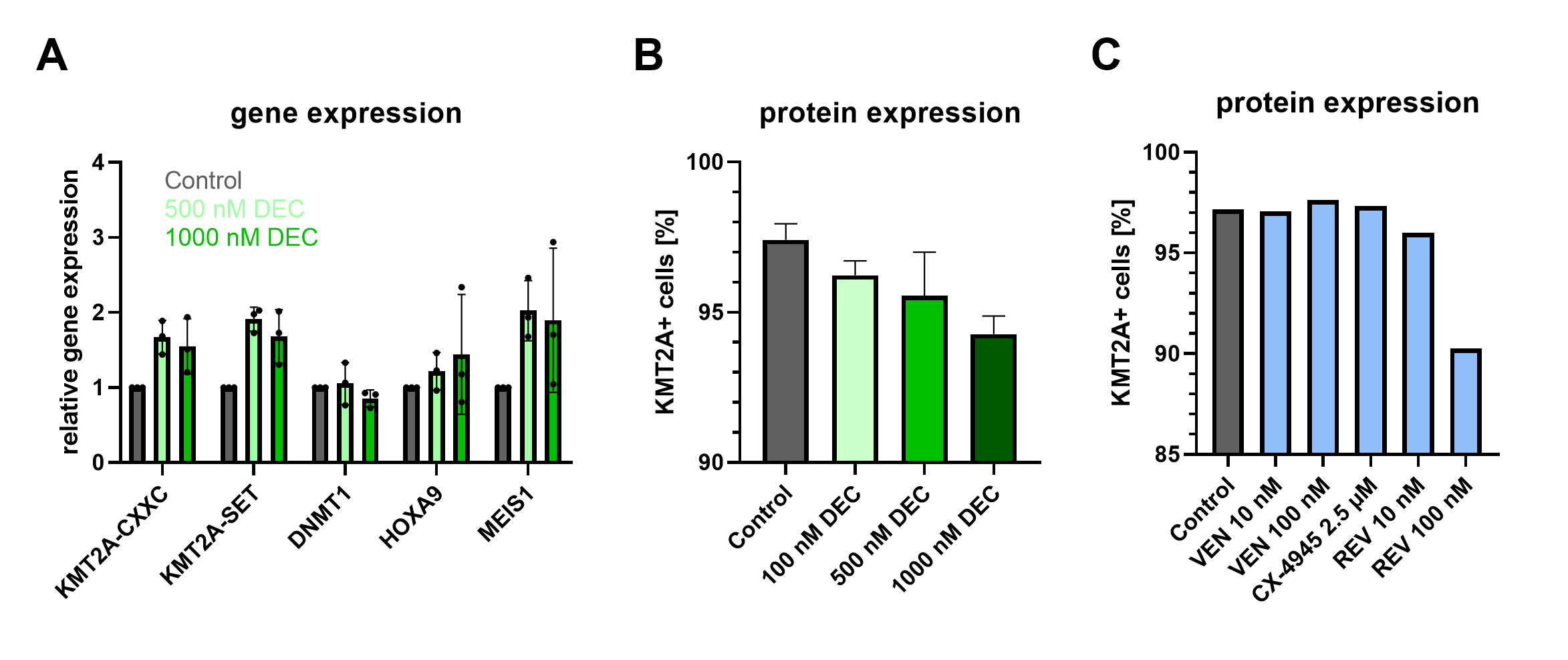

Supplement: Supplementary file 1 — Fig. S1. Concentration‐dependent effects of DEC on gene, and KMT2A protein expression in SEM cells. Fig. S2. Basal gene expression in cell lines (SEM, RS4;11, REH, NALM‐6), primary samples (four‐digit numbers), and five healthy donor B‐cells. Fig. S3. Gene and protein expression of DNMT1, KMT2A, HOXA9, and MEIS1. Fig. S4. Gene and protein expression of DNMT1, KMT2A, HOXA9, and MEIS1. Fig. S5. Influence of siRNA‐mediated transcriptional silencing of KMT2A and DNMT1 on cell proliferation, and decitabine response in SEM and NALM‐6 cells. Fig. S6. Probe‐based gene expression analysis of CDKN2C following 72 h 1 μm DEC incubation. Fig. S7. DEC‐mediated effects on HOXA9, and MEIS1 in xenograft model systems. Fig. S8. Concentration‐dependent effects of menin inhibitor revumenib (REV) on acute leukemia cell lines. Fig. S9. Effects of simultaneous 72 h DEC (100 nm), and REV (10 nm) incubation on methyltransferase‐mediated signaling pathways. [file MOL2-19-1404-s001.zip › mol213792-sup-0001-FigureS1.tif]

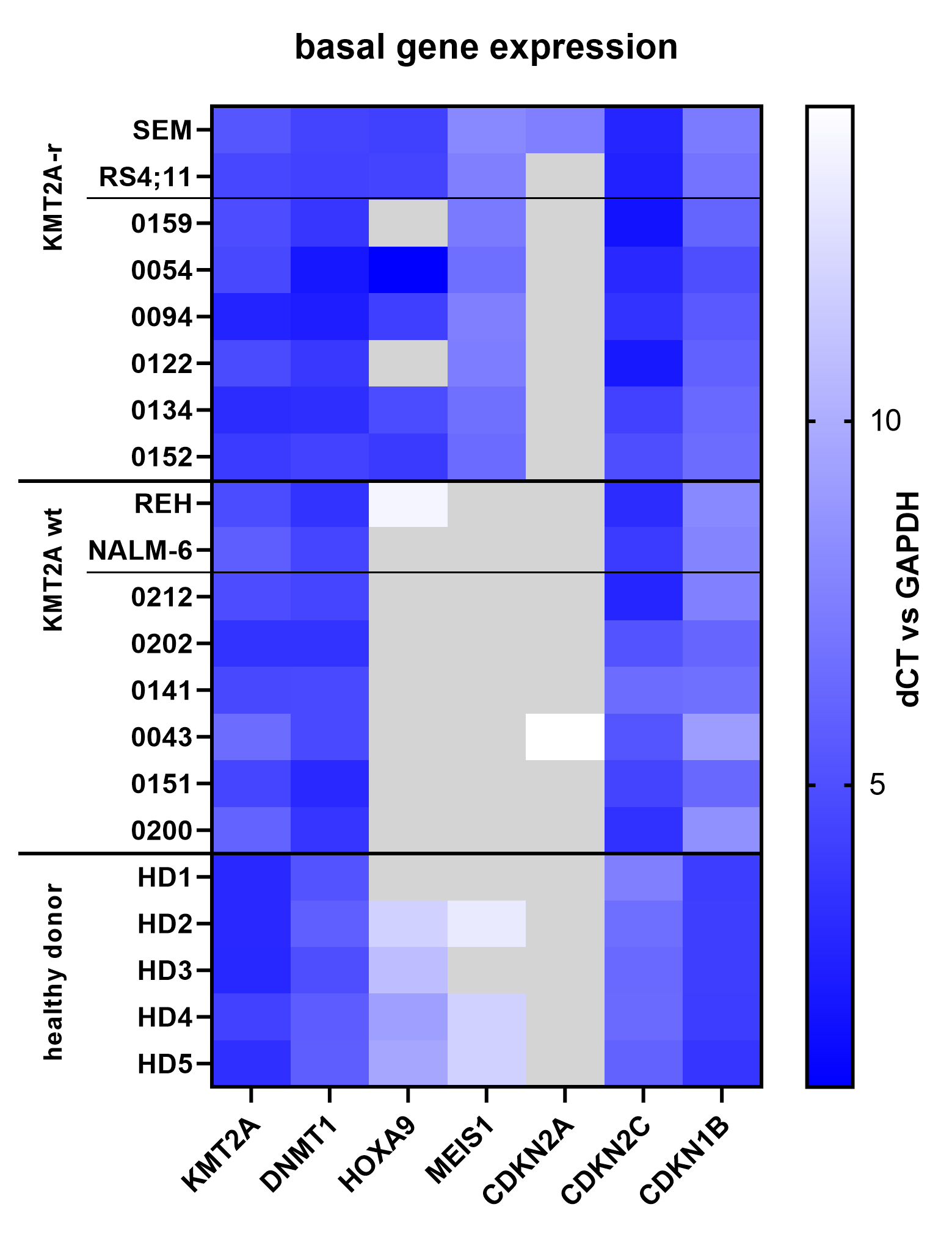

Supplement: Supplementary file 1 — Fig. S1. Concentration‐dependent effects of DEC on gene, and KMT2A protein expression in SEM cells. Fig. S2. Basal gene expression in cell lines (SEM, RS4;11, REH, NALM‐6), primary samples (four‐digit numbers), and five healthy donor B‐cells. Fig. S3. Gene and protein expression of DNMT1, KMT2A, HOXA9, and MEIS1. Fig. S4. Gene and protein expression of DNMT1, KMT2A, HOXA9, and MEIS1. Fig. S5. Influence of siRNA‐mediated transcriptional silencing of KMT2A and DNMT1 on cell proliferation, and decitabine response in SEM and NALM‐6 cells. Fig. S6. Probe‐based gene expression analysis of CDKN2C following 72 h 1 μm DEC incubation. Fig. S7. DEC‐mediated effects on HOXA9, and MEIS1 in xenograft model systems. Fig. S8. Concentration‐dependent effects of menin inhibitor revumenib (REV) on acute leukemia cell lines. Fig. S9. Effects of simultaneous 72 h DEC (100 nm), and REV (10 nm) incubation on methyltransferase‐mediated signaling pathways. [file MOL2-19-1404-s001.zip › mol213792-sup-0002-FigureS2.tif]

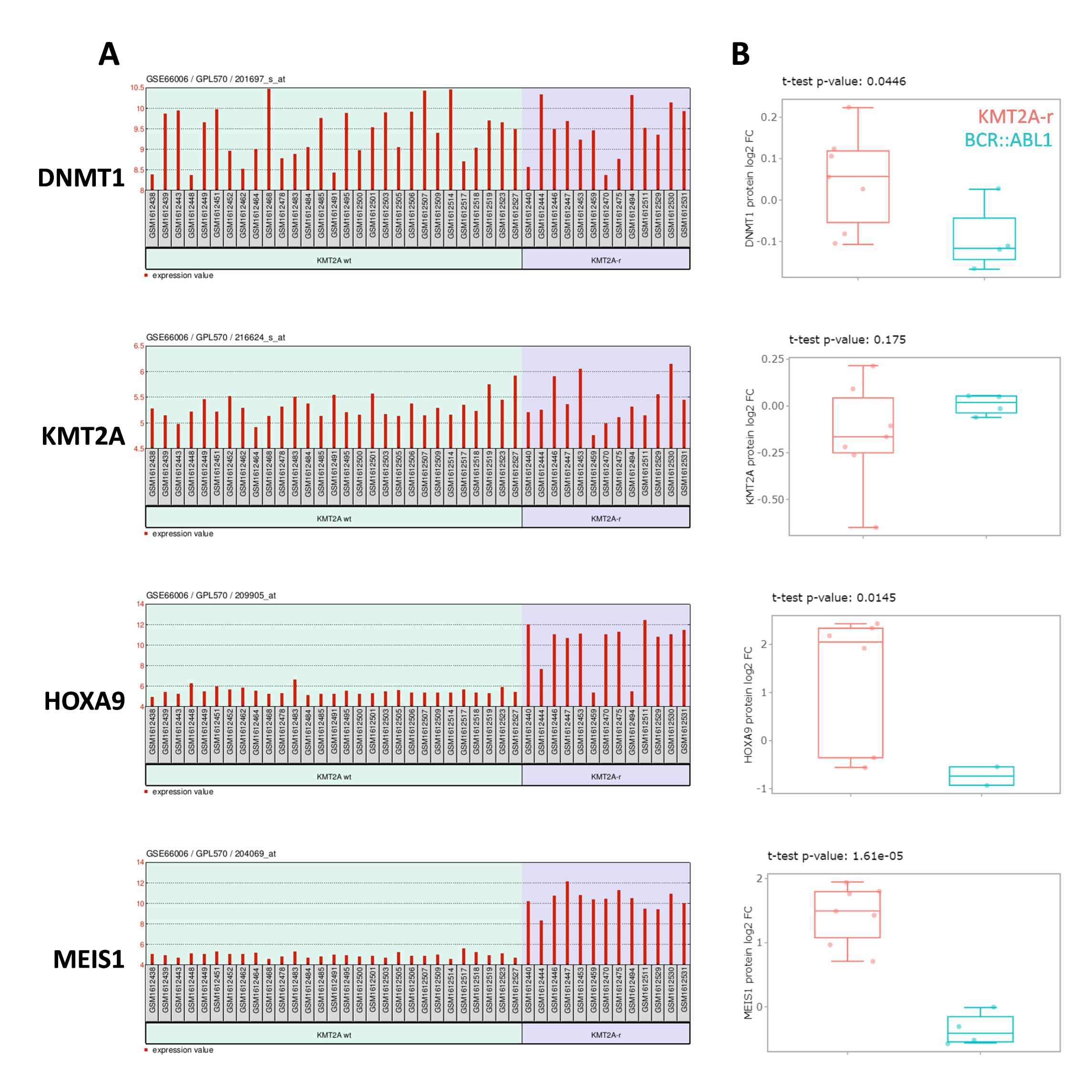

Supplement: Supplementary file 1 — Fig. S1. Concentration‐dependent effects of DEC on gene, and KMT2A protein expression in SEM cells. Fig. S2. Basal gene expression in cell lines (SEM, RS4;11, REH, NALM‐6), primary samples (four‐digit numbers), and five healthy donor B‐cells. Fig. S3. Gene and protein expression of DNMT1, KMT2A, HOXA9, and MEIS1. Fig. S4. Gene and protein expression of DNMT1, KMT2A, HOXA9, and MEIS1. Fig. S5. Influence of siRNA‐mediated transcriptional silencing of KMT2A and DNMT1 on cell proliferation, and decitabine response in SEM and NALM‐6 cells. Fig. S6. Probe‐based gene expression analysis of CDKN2C following 72 h 1 μm DEC incubation. Fig. S7. DEC‐mediated effects on HOXA9, and MEIS1 in xenograft model systems. Fig. S8. Concentration‐dependent effects of menin inhibitor revumenib (REV) on acute leukemia cell lines. Fig. S9. Effects of simultaneous 72 h DEC (100 nm), and REV (10 nm) incubation on methyltransferase‐mediated signaling pathways. [file MOL2-19-1404-s001.zip › mol213792-sup-0003-FigureS3.tif]

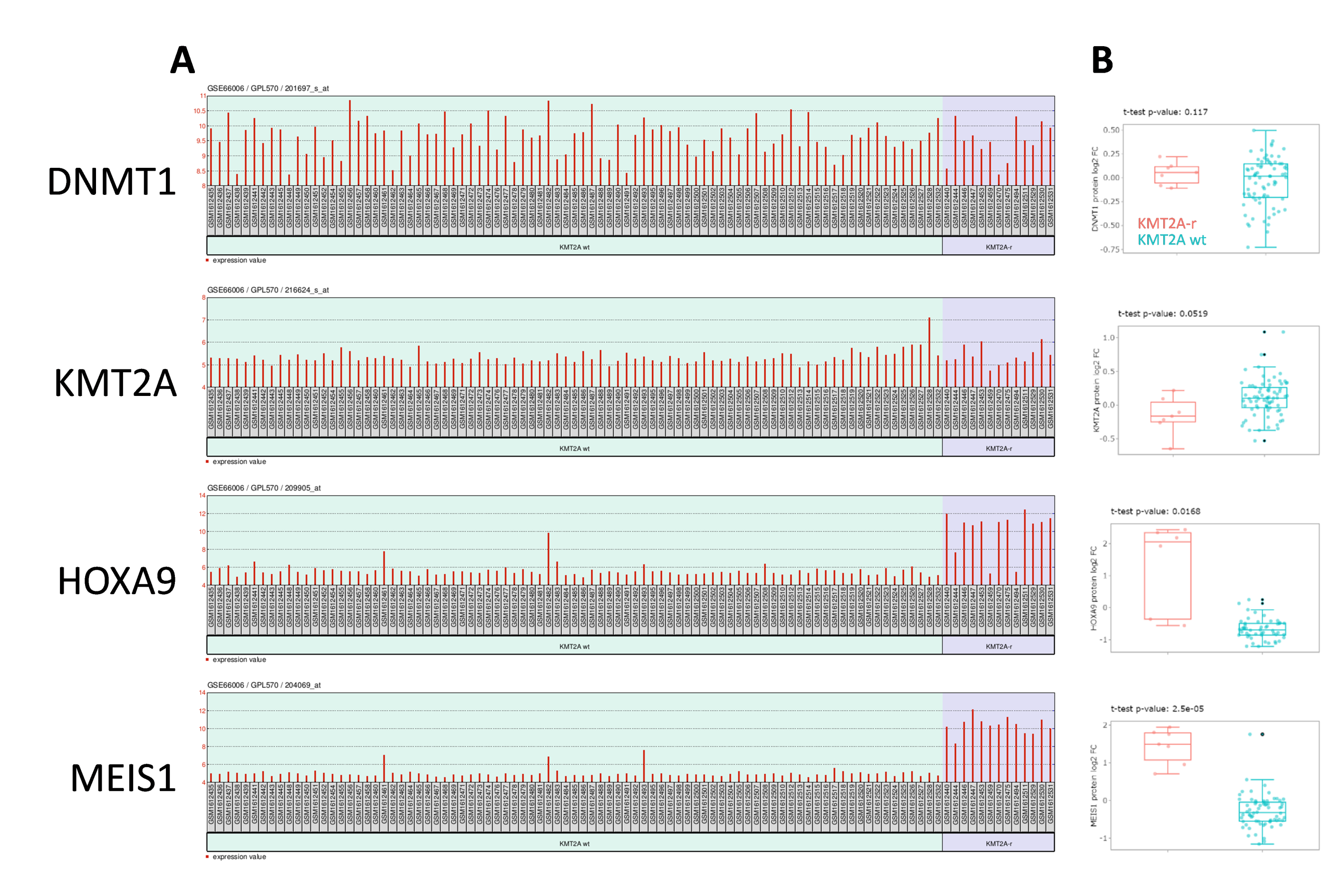

Supplement: Supplementary file 1 — Fig. S1. Concentration‐dependent effects of DEC on gene, and KMT2A protein expression in SEM cells. Fig. S2. Basal gene expression in cell lines (SEM, RS4;11, REH, NALM‐6), primary samples (four‐digit numbers), and five healthy donor B‐cells. Fig. S3. Gene and protein expression of DNMT1, KMT2A, HOXA9, and MEIS1. Fig. S4. Gene and protein expression of DNMT1, KMT2A, HOXA9, and MEIS1. Fig. S5. Influence of siRNA‐mediated transcriptional silencing of KMT2A and DNMT1 on cell proliferation, and decitabine response in SEM and NALM‐6 cells. Fig. S6. Probe‐based gene expression analysis of CDKN2C following 72 h 1 μm DEC incubation. Fig. S7. DEC‐mediated effects on HOXA9, and MEIS1 in xenograft model systems. Fig. S8. Concentration‐dependent effects of menin inhibitor revumenib (REV) on acute leukemia cell lines. Fig. S9. Effects of simultaneous 72 h DEC (100 nm), and REV (10 nm) incubation on methyltransferase‐mediated signaling pathways. [file MOL2-19-1404-s001.zip › mol213792-sup-0004-FigureS4.tif]

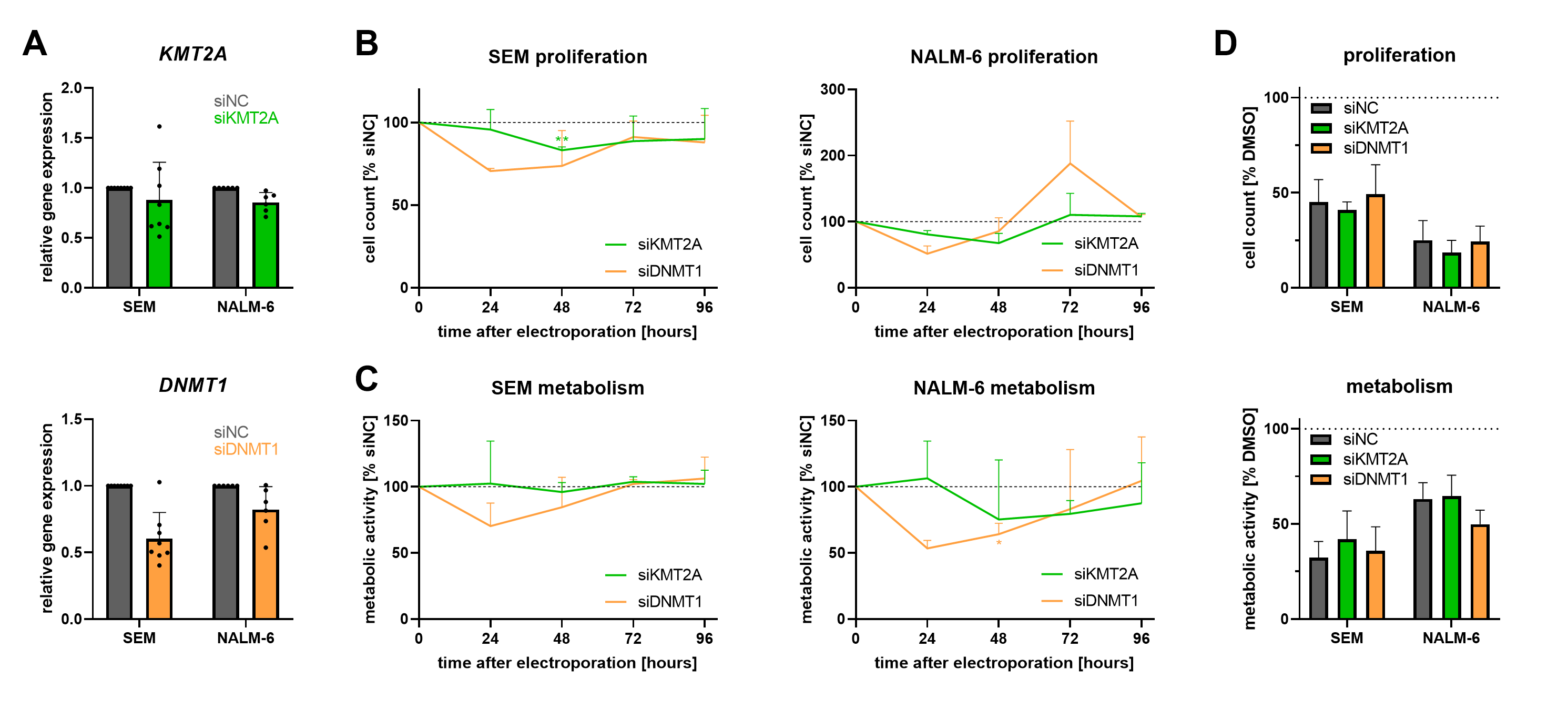

Supplement: Supplementary file 1 — Fig. S1. Concentration‐dependent effects of DEC on gene, and KMT2A protein expression in SEM cells. Fig. S2. Basal gene expression in cell lines (SEM, RS4;11, REH, NALM‐6), primary samples (four‐digit numbers), and five healthy donor B‐cells. Fig. S3. Gene and protein expression of DNMT1, KMT2A, HOXA9, and MEIS1. Fig. S4. Gene and protein expression of DNMT1, KMT2A, HOXA9, and MEIS1. Fig. S5. Influence of siRNA‐mediated transcriptional silencing of KMT2A and DNMT1 on cell proliferation, and decitabine response in SEM and NALM‐6 cells. Fig. S6. Probe‐based gene expression analysis of CDKN2C following 72 h 1 μm DEC incubation. Fig. S7. DEC‐mediated effects on HOXA9, and MEIS1 in xenograft model systems. Fig. S8. Concentration‐dependent effects of menin inhibitor revumenib (REV) on acute leukemia cell lines. Fig. S9. Effects of simultaneous 72 h DEC (100 nm), and REV (10 nm) incubation on methyltransferase‐mediated signaling pathways. [file MOL2-19-1404-s001.zip › mol213792-sup-0005-FigureS5.tif]

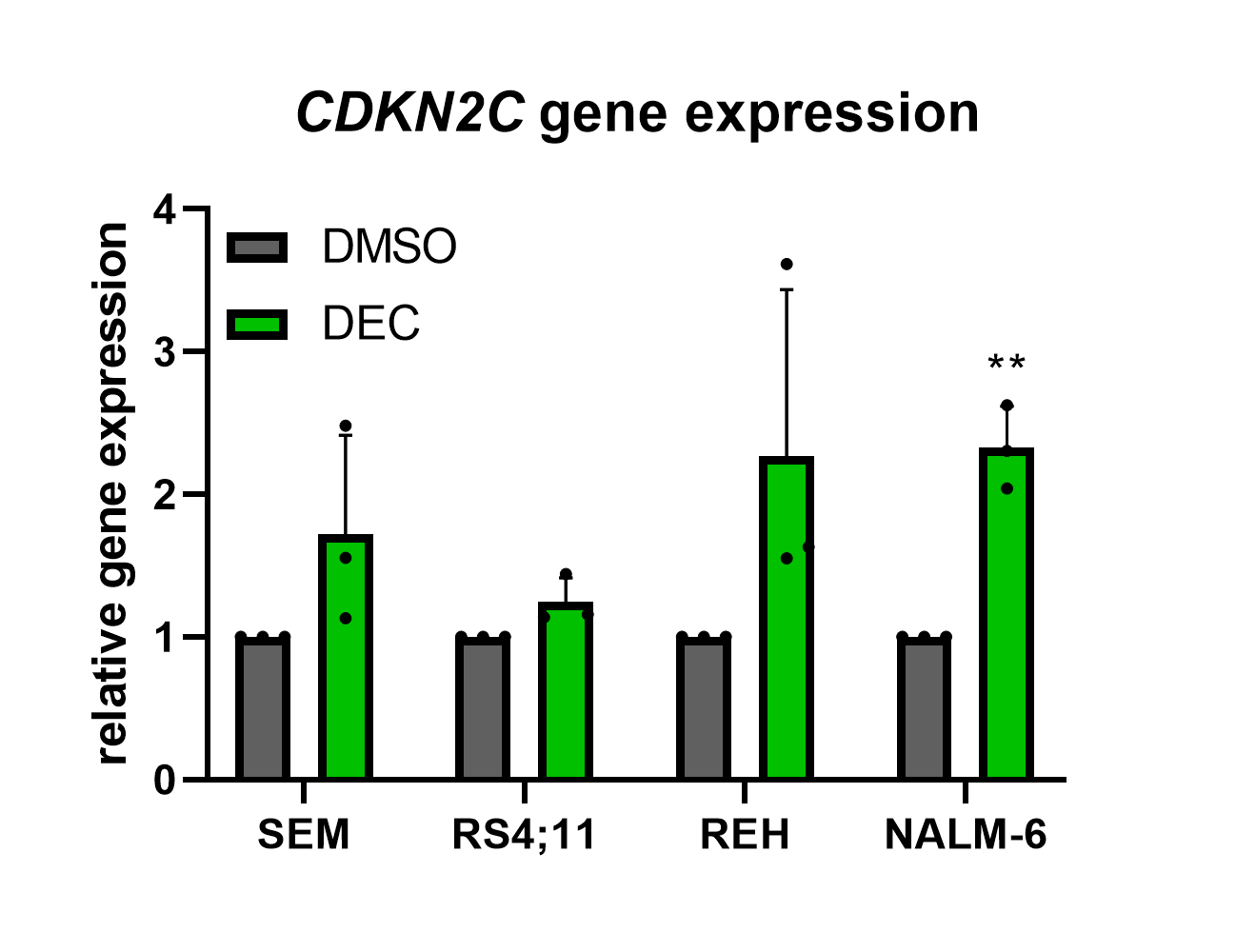

Supplement: Supplementary file 1 — Fig. S1. Concentration‐dependent effects of DEC on gene, and KMT2A protein expression in SEM cells. Fig. S2. Basal gene expression in cell lines (SEM, RS4;11, REH, NALM‐6), primary samples (four‐digit numbers), and five healthy donor B‐cells. Fig. S3. Gene and protein expression of DNMT1, KMT2A, HOXA9, and MEIS1. Fig. S4. Gene and protein expression of DNMT1, KMT2A, HOXA9, and MEIS1. Fig. S5. Influence of siRNA‐mediated transcriptional silencing of KMT2A and DNMT1 on cell proliferation, and decitabine response in SEM and NALM‐6 cells. Fig. S6. Probe‐based gene expression analysis of CDKN2C following 72 h 1 μm DEC incubation. Fig. S7. DEC‐mediated effects on HOXA9, and MEIS1 in xenograft model systems. Fig. S8. Concentration‐dependent effects of menin inhibitor revumenib (REV) on acute leukemia cell lines. Fig. S9. Effects of simultaneous 72 h DEC (100 nm), and REV (10 nm) incubation on methyltransferase‐mediated signaling pathways. [file MOL2-19-1404-s001.zip › mol213792-sup-0006-FigureS6.tif]

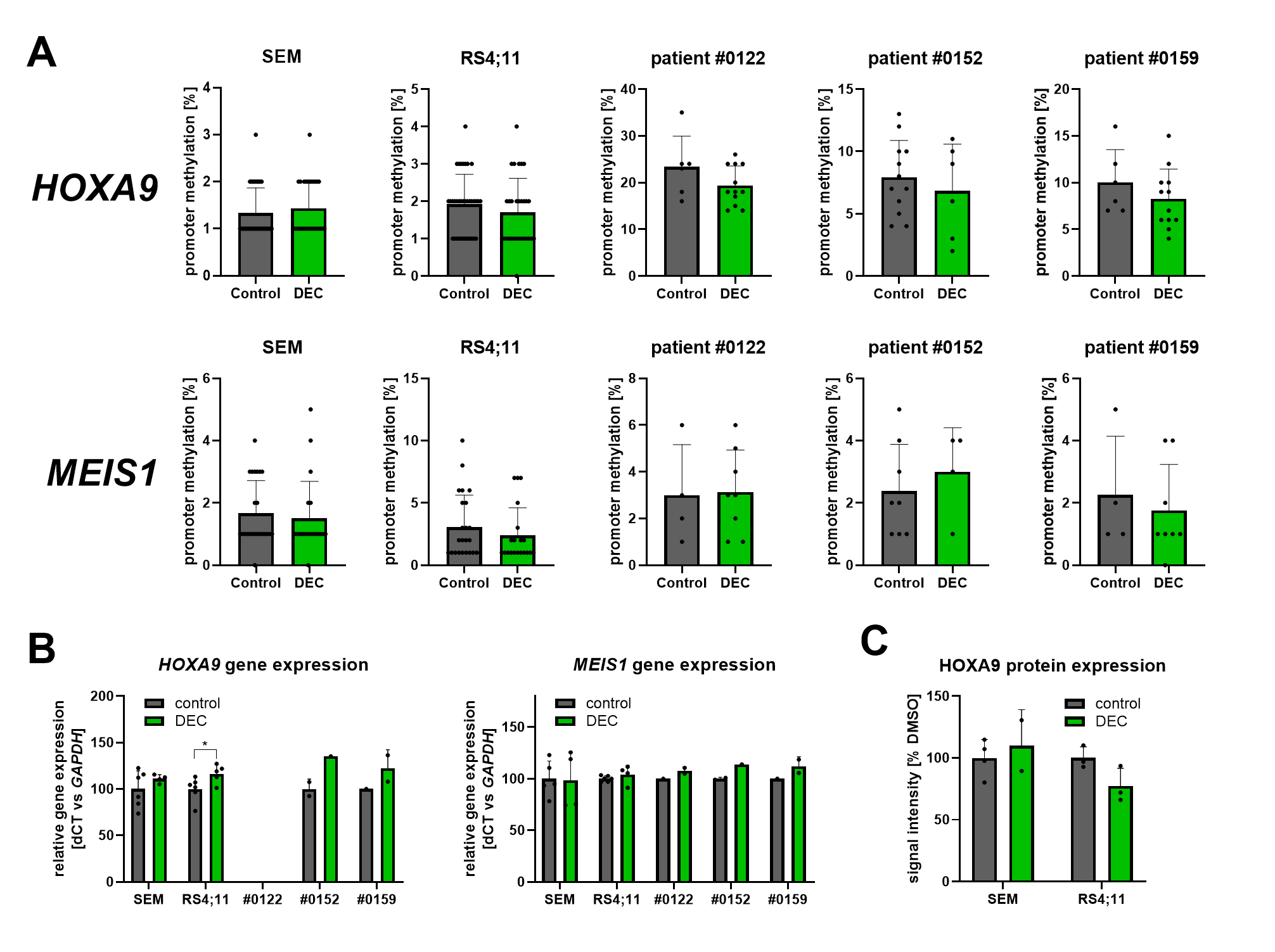

Supplement: Supplementary file 1 — Fig. S1. Concentration‐dependent effects of DEC on gene, and KMT2A protein expression in SEM cells. Fig. S2. Basal gene expression in cell lines (SEM, RS4;11, REH, NALM‐6), primary samples (four‐digit numbers), and five healthy donor B‐cells. Fig. S3. Gene and protein expression of DNMT1, KMT2A, HOXA9, and MEIS1. Fig. S4. Gene and protein expression of DNMT1, KMT2A, HOXA9, and MEIS1. Fig. S5. Influence of siRNA‐mediated transcriptional silencing of KMT2A and DNMT1 on cell proliferation, and decitabine response in SEM and NALM‐6 cells. Fig. S6. Probe‐based gene expression analysis of CDKN2C following 72 h 1 μm DEC incubation. Fig. S7. DEC‐mediated effects on HOXA9, and MEIS1 in xenograft model systems. Fig. S8. Concentration‐dependent effects of menin inhibitor revumenib (REV) on acute leukemia cell lines. Fig. S9. Effects of simultaneous 72 h DEC (100 nm), and REV (10 nm) incubation on methyltransferase‐mediated signaling pathways. [file MOL2-19-1404-s001.zip › mol213792-sup-0007-FigureS7.tif]

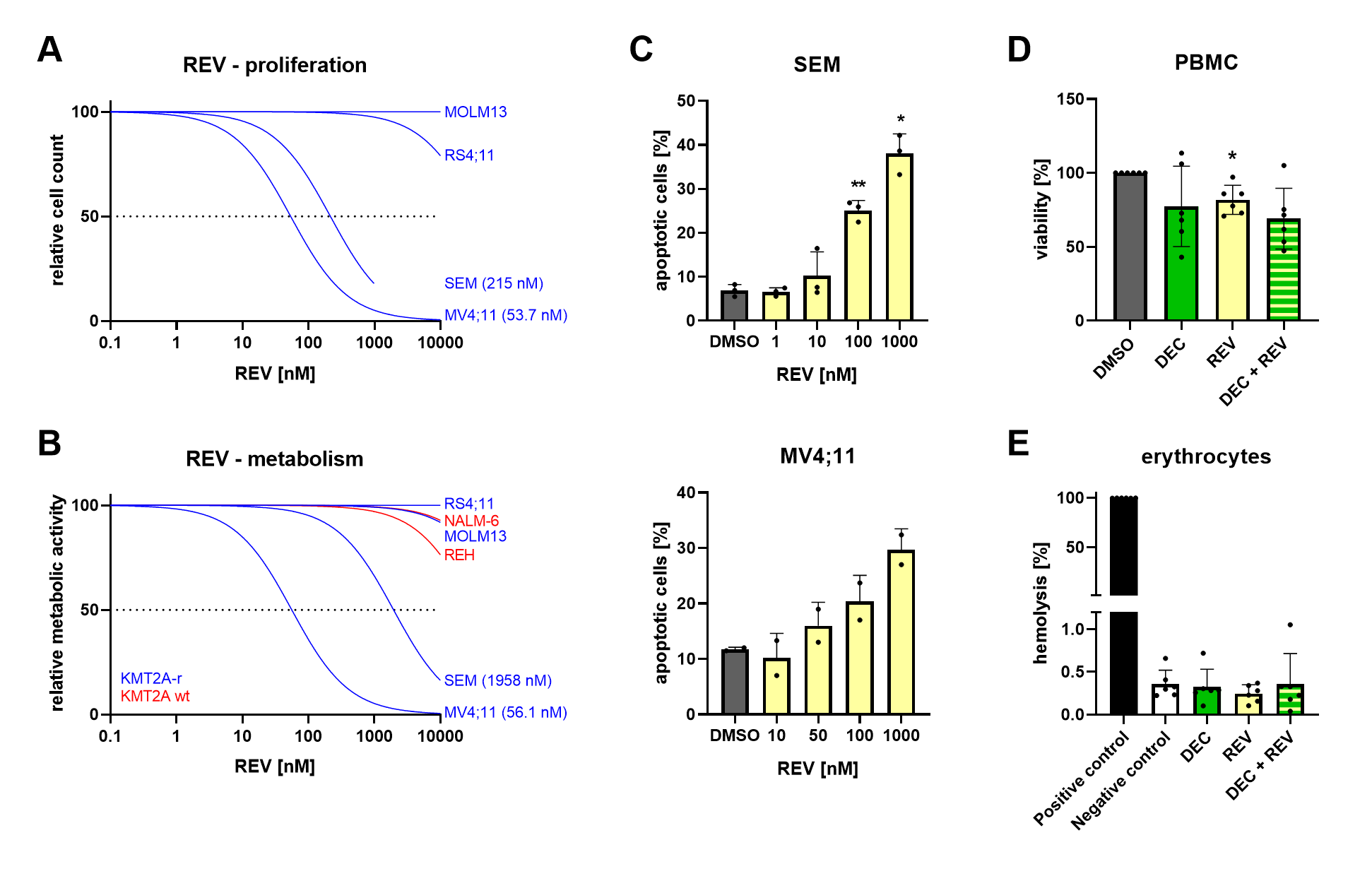

Supplement: Supplementary file 1 — Fig. S1. Concentration‐dependent effects of DEC on gene, and KMT2A protein expression in SEM cells. Fig. S2. Basal gene expression in cell lines (SEM, RS4;11, REH, NALM‐6), primary samples (four‐digit numbers), and five healthy donor B‐cells. Fig. S3. Gene and protein expression of DNMT1, KMT2A, HOXA9, and MEIS1. Fig. S4. Gene and protein expression of DNMT1, KMT2A, HOXA9, and MEIS1. Fig. S5. Influence of siRNA‐mediated transcriptional silencing of KMT2A and DNMT1 on cell proliferation, and decitabine response in SEM and NALM‐6 cells. Fig. S6. Probe‐based gene expression analysis of CDKN2C following 72 h 1 μm DEC incubation. Fig. S7. DEC‐mediated effects on HOXA9, and MEIS1 in xenograft model systems. Fig. S8. Concentration‐dependent effects of menin inhibitor revumenib (REV) on acute leukemia cell lines. Fig. S9. Effects of simultaneous 72 h DEC (100 nm), and REV (10 nm) incubation on methyltransferase‐mediated signaling pathways. [file MOL2-19-1404-s001.zip › mol213792-sup-0008-FigureS8.tif]

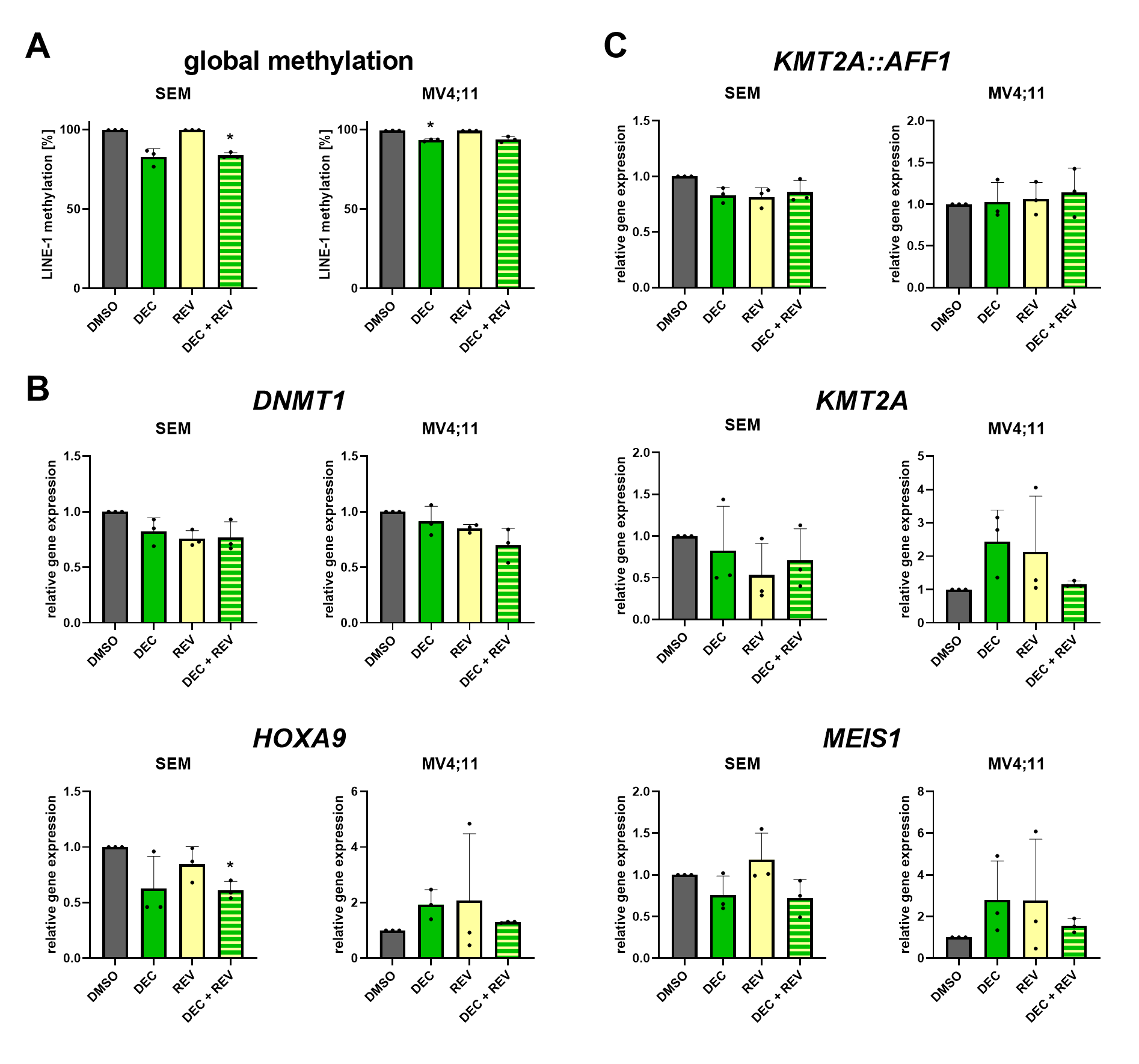

Supplement: Supplementary file 1 — Fig. S1. Concentration‐dependent effects of DEC on gene, and KMT2A protein expression in SEM cells. Fig. S2. Basal gene expression in cell lines (SEM, RS4;11, REH, NALM‐6), primary samples (four‐digit numbers), and five healthy donor B‐cells. Fig. S3. Gene and protein expression of DNMT1, KMT2A, HOXA9, and MEIS1. Fig. S4. Gene and protein expression of DNMT1, KMT2A, HOXA9, and MEIS1. Fig. S5. Influence of siRNA‐mediated transcriptional silencing of KMT2A and DNMT1 on cell proliferation, and decitabine response in SEM and NALM‐6 cells. Fig. S6. Probe‐based gene expression analysis of CDKN2C following 72 h 1 μm DEC incubation. Fig. S7. DEC‐mediated effects on HOXA9, and MEIS1 in xenograft model systems. Fig. S8. Concentration‐dependent effects of menin inhibitor revumenib (REV) on acute leukemia cell lines. Fig. S9. Effects of simultaneous 72 h DEC (100 nm), and REV (10 nm) incubation on methyltransferase‐mediated signaling pathways. [file MOL2-19-1404-s001.zip › mol213792-sup-0009-FigureS9.tif]
