# Supplementary material for: KMT2A degradation is observed in decitabine‐responsive acute lymphoblastic leukemia cells
Source: Mol Oncol. 2025 Jan 4;19(5):1404–21. doi: 10.1002/1878-0261.13792 (PMC12077275; doi:10.1002/1878-0261.13792)
Supplement: Supplementary file 2 — Table S1. Western blot antibodies. [file MOL2-19-1404-s004.docx]

Table S1: Western blot antibodies.

| Target | Brand | Cat-No | Solvent | Dilution |
| --- | --- | --- | --- | --- |
| H3K4me3 | Cell Signaling | 9751 | TBST + 5% BSA | 1:1000 |
| DNMT1 | Proteintech | 24206-1-AP | TBST + 5% skim milk | 1:300 |
| KMT2A | Cell Signaling | 14197 | TBST + 5% BSA | 1:1000 |
| HOXA9 | Proteintech | 18501-1-AP | 1:5 LI-COR blocking buffer:PBST | 1:500 |
| MEIS1/2 | Cell Signaling | 12744 | TBST + 5% BSA | 1:1000 |
| p16 | Proteintech | 10883-1-AP | TBST + 5% skim milk | 1:500 |
| p27 | Cell Signaling | 3686 | TBST + 5% skim milk | 1:500 |
| GAPDH | Invitrogen | MA1-16757 | TBST + 5% BSA | 1:10,000 |
