# Supplementary material for: KMT2A degradation is observed in decitabine‐responsive acute lymphoblastic leukemia cells
Source: Mol Oncol. 2025 Jan 4;19(5):1404–21. doi: 10.1002/1878-0261.13792 (PMC12077275; doi:10.1002/1878-0261.13792)
Supplement: Supplementary file 3 — Table S2. Primers for gene expression analyses. [file MOL2-19-1404-s003.docx]

Table S2: Primers for gene expression analyses.

| Target | Forward primer | Reverse primer | Quencher |
| --- | --- | --- | --- |
| KMT2A-SET | TGATGCAGGTGAGATGGTGA | AGTGATTGATGAAGCGTGCA | CCATCCAGACTGACAAGCGGGAAAAGT |
| KMT2A-CXXC | ACCCTGAGTGCCTTACCATG | TGGGGTGCCTTGTTTCTAGT | GAACCTCTTGCTCCACCCATCAAACCA |
| DNMT1 | ACTGGCTTTGATGGAGGTGA | ACCGTGGTCTCGATCTTGTT | AAGATTGTGGTGGAGTTCCTGCAGAGC |
| HOXA9 | ACGCTTGACACTCACACTTTG | TTCTCCAGTTCCAGGGTCTG | CTCGGAAAAAGCGGTGCCCCTATACAA |
| MEIS1 | CGGCATCTACTCGTTCAGGA | AGCCACGCCCTCATGATATT | AGCGTCACAAAAAGCGTGGCATCTTTC |
| CDKN2A | CCGAATAGTTACGGTCGGAG | CTCCTCAGCCAGGTCCAC | CTGCCCATCATCATGACCTGGATC |
| CDKN2C | GATTTGGAAGGACTGCGCTG | CATCAGCTTGAAACTCCAGCA | AGGTGCTAATCCCGATTTGAAAGACCGA |
| CDKN1B | CTCTGAGGACACGCATTTGG | CTCCACAGAACCGGCATTTG | GCGACCTGCAACCGACGATTCTTCTAC |
| KMT2A::AFF1 | CCGCCCAAGTATCCCTGTAA | GCTCAGCTGTACTAGGCGTA | TGGCCGCCTCCTTTGACAGCA |
| GAPDH | TCACCAGGGCTGCTTTTAAC | GGGTGGAATCATATTGGAACA | TGCCATCAATGACCCCTTCATTG |
