# Supplementary material for: KMT2A degradation is observed in decitabine‐responsive acute lymphoblastic leukemia cells
Source: Mol Oncol. 2025 Jan 4;19(5):1404–21. doi: 10.1002/1878-0261.13792 (PMC12077275; doi:10.1002/1878-0261.13792)
Supplement: Supplementary file 4 — Table S3. Patient characteristics. [file MOL2-19-1404-s002.docx]

Table S3: Patient characteristics

| LabID | Subtype | Sex | Age | Diagnosis | Sample type | Cytogenetics |
| --- | --- | --- | --- | --- | --- | --- |
| 0054 | Pro B-ALL | F | 70 | Initial | Bone marrow | 46,XX,t(4;11)(q21;q23) |
| 0094 | Mature B-ALL | F | 85 | Relapse | Blood | 46,XX nuc ish(MLLtel,MLLcen)x2(MlltelsepMLLcenx1) |
| 0122 | Pro B-ALL | M | 47 | Initial | Blood | 46,XY,t(4;11)(q21;q23)[13]/47,XY,+X,t(4;11)(q21;q23),-9, +21[1]/ 48,XY,+X,t(4;11)(q21;q23),+21[2] |
| 0134 | Pro B-ALL | M | 43 | Initial | Bone marrow | 46,XY, FISH: del 11q23(MLL) and +22 |
| 0152 | Pro B-ALL, biphenotypic | F | 52 | Initial | Blood | 46,XX,t(4;11)(q21;q23) |
| 0159 | B-ALL | F | 74 | Initial | Bone marrow | 46,XX,t(4;11)(q21;q23) |
| 0043 | Common B-ALL | F | 40 | Initial | Blood | 47,XX,+8,t(9;22)(q34;q11),der(9)t(9;22)(q34;q11) |
| 0141 | Common B-ALL, biphenotypic | M | 43 | Initial | Bone marrow | 46,XY,t(9;22)(q34;q11.2),+21,der(22)t(9;22)(q34;q11.2)[6]/ 46,XY,t(9;22)(q34;q11.2)[21] |
| 0151 | B-ALL | M | 84 | Initial | Blood | FISH: bcr/abl positive, del(9)(p21) (=p16) |
| 0200 | B-ALL | F | 37 | Initial | Blood | 46,XX,t(9;22)(q34;q1.1) |
| 0202 | Common B-ALL | M | 36 | Initial | Bone marrow | 46,XY |
| 0212 | Common B-ALL | F | 62 | Relapse | Bone marrow | 47,XX,t(9;22)(q34;q11),+der(22)t(9;22)(q34;q11) [9] |
