# Supplementary material for: KMT2A degradation is observed in decitabine‐responsive acute lymphoblastic leukemia cells
Source: Mol Oncol. 2025 Jan 4;19(5):1404–21. doi: 10.1002/1878-0261.13792 (PMC12077275; doi:10.1002/1878-0261.13792)
Supplement: Supplementary file 5 — Table S4. Bliss synergy values for combined decitabine (DEC), and revumenib (REV) incubation. [file MOL2-19-1404-s005.docx]

Table S4: Bliss synergy values for combined DEC and REV incubation

|  | Proliferation | | | Metabolic activity | | | apoptosis |
| --- | --- | --- | --- | --- | --- | --- | --- |
|  | DEC + REV | DEC > REV | REV > DEC | DEC + REV | DEC > REV | REV > DEC | DEC + REV |
| SEM | 0,131 | 0,040 | 0,014 | 0,277 | 0,275 | 0,147 | 0,017 |
| MV4;11 | 0,046 | x | x | 0,024 | x | x | -0,122 |

Positive values indicate synergy while negative values suggest antagonism
